# Multimorbidity patterns and the subsequent risk of albuminuria: findings from the Stockholm Creatinine Measurements (SCREAM) project

**DOI:** 10.1186/s12916-026-04772-5

**Published:** 2026-03-24

**Authors:** Giorgi Beridze, Patrick B. Mark, Michael K. Sullivan, Heather Walker, Shigeru Tanaka, Anne-Laure Faucon, Davide L. Vetrano, Amaia Calderón-Larrañaga, Juan-Jesus Carrero

**Affiliations:** 1 Aging Research Center, Department of Neurobiology, Care Sciences and Society, Karolinska Institutet and Stockholm University, Stockholm, Sweden; 2 School of Cardiovascular and Metabolic Health, University of Glasgow, Glasgow, Scotland; 3 Renal and Transplant Unit, Queen Elizabeth University Hospital, Glasgow, Scotland; 4 Department of Medical Epidemiology and Biostatistics, Karolinska Institutet, Stockholm, Sweden; 5 Department of Medicine and Clinical Science, Graduate School of Medical Sciences, Kyushu University, Fukuoka, Japan; 6 Stockholm Gerontology Research Center, Stockholm, Sweden; 7 Division of Renal Medicine, Department of Clinical Sciences, Danderyd Hospital, Danderyd, Sweden

**Keywords:** Multimorbidity, Albuminuria, Kidney diseases, Risk factors, Cohort studies

## Abstract

**Background:**

Chronic conditions often cluster together, forming distinct multimorbidity patterns. We aimed to explore how such patterns are associated with the risk of albuminuria.

**Methods:**

We utilized the Stockholm Creatinine Measurements (SCREAM) project, comprising 675,570 adults undergoing outpatient albuminuria testing in Stockholm, Sweden. Disease patterns were derived in adults without albuminuria at baseline, stratified by age (18–64, 65–74, ≥ 75 years). Associations with incident albuminuria (albumin-creatinine ratio > 30 mg/g) and macroalbuminuria (> 300 mg/g) were examined using Cox and Fine-Gray competing risk models.

**Results:**

We identified four multimorbidity patterns in younger participants (18–64), six in the 65–74 group, and seven among those aged ≥ 75. Across all age groups, most patterns—including cardiovascular, mental health, and eye patterns—were consistently associated with elevated albuminuria risk compared to those without multimorbidity. In the 65–74 stratum, the cardiovascular pattern had the highest risk compared to those without multimorbidity (HR 3.09: 95% CI: 2.85–3.34). Among those aged ≥ 75, almost all identified patterns showed higher risk, with those in the vascular pattern being at highest risk (HR: 2.41, 95% CI: 2.08–2.80). The dementia pattern showed no significant association. High-burden patterns in older participants with numerous chronic conditions (e.g., multisystem and cardiovascular patterns) were at increased risk, but this was attenuated after accounting for the competing risk of death. The 5- to 6-year cumulative incidence of albuminuria exceeded 10–14% in the highest-risk patterns, 5–9% higher than for those without multimorbidity. In interaction analyses, reduced estimated glomerular filtration rate amplified risk among younger individuals. Females generally had lower risk, except with mental health patterns in younger ages or cardiovascular multimorbidity in those 65 to 74.

**Conclusions:**

Multimorbidity patterns, including those characterized by non-traditional CKD risk factors, can help identify individuals at elevated albuminuria risk. Targeted screening of these groups may enable preventive strategies to slow or prevent kidney damage.

**Supplementary Information:**

The online version contains supplementary material available at 10.1186/s12916-026-04772-5.

## Background

Multimorbidity, defined as the co-occurrence of two or more chronic conditions, is a growing global health challenge, affecting approximately one in four adults in the general population and more than two-thirds of older adults [1]. Chronic conditions rarely occur at random but instead cluster into distinct and replicable patterns due to shared risk factors, pathophysiological mechanisms, and disease interactions [2]. The study of these patterns—referred to as associative multimorbidity—has been increasingly recognized as a key research priority [3]. Several multimorbidity patterns have been consistently identified across populations [4, 5], and such patterns have been linked to important health outcomes, such as disability [6], frailty [7], and unplanned hospitalizations [8].

Kidney function decline is a common consequence of multimorbidity and is closely linked to the increasing prevalence of chronic kidney disease (CKD), which in turn is associated with worsening health outcomes and increased mortality [9]. Albuminuria, defined as an abnormal excretion of albumin in the urine, is an early marker of kidney damage that reflects structural injury of the glomerular filtration barrier. Albuminuria is a strong independent predictor of future cardiovascular events [10], subsequent kidney function decline [11], kidney failure [12], and mortality [12]. Several individual chronic diseases, including hypertension, diabetes, and cardiovascular disease, have been implicated in the pathophysiology of albuminuria [13]. However, the extent to which broader multimorbidity influences albuminuria risk remains unclear.

Previous studies examining the relationship between multimorbidity and kidney function have primarily focused on either estimated glomerular filtration rate (eGFR) decline or end-stage kidney disease (ESKD) as an outcome [14–17], which represent late stages of disease progression. While these studies provide valuable insights, none have considered albuminuria as an outcome, despite its clinical significance in detecting early kidney damage and its close association with adverse outcomes. Identifying multimorbidity patterns associated with albuminuria could improve risk stratification, identifying populations where targeted screening and early use of anti-proteinuric medications may be helpful. In addition, it can also provide insights into underlying etiological mechanisms.

This study aimed to identify multimorbidity patterns associated with an increased risk of incident albuminuria within a large healthcare system cohort.

## Methods

### Data source

This longitudinal study is based on data from the Stockholm Creatinine Measurements Project (SCREAM), a healthcare utilization cohort encompassing all individuals residing in Stockholm County, Sweden [18]. SCREAM is a complex data linkage of national and regional registries that captures complete healthcare data of all residents in Stockholm County between 2006 and 2021, including laboratory tests, sociodemographics, mortality and medications. The registries used in the present study include: the Stockholm Regional Healthcare Data Warehouse (VAL), which contains information on diagnoses from visits across all levels of healthcare; the National Prescribed Drug Register, which contains information on dispensed prescription medication coded using the Anatomical Therapeutic Chemical (ATC) classification; the Swedish Renal Registry, which captures all patients starting kidney replacement therapy (dialysis or kidney transplantation) and the National Cause of Death register, which includes information on dates and causes of death.

### Participant selection

The participant selection process is outlined in Additional file 1: Fig S1. Out of 1,202,251 individuals with an albuminuria test available in SCREAM, we excluded those with tests only from inpatient settings, those under the age of 18 at the time of the test, and those without a concurrent eGFR measurement in the 12 months preceding their test, leaving a population of 834,762 individuals. We further excluded participants with pre-existing albuminuria (> 30 mg/g) or history of kidney replacement therapy at their first eligible measurement, those who were non-resident at the time of the test, and those who died or emigrated on the day of the test, leaving a study population of 675,570 individuals. Information on age and sex was obtained at the time of the first eligible test date. These participants were divided into three age strata: 18 to 65 years old (young to middle-aged adults), 65–74 years old (youngest-old), and 75 and above (middle- and oldest-old), based on previous evidence demonstrating that both the prevalence and composition of multimorbidity vary substantially across different age groups [4].

### Multimorbidity

We identified 60 chronic conditions based on a previously described methodology [19]. In short, a multidisciplinary team identified and mapped relevant diagnostic ICD-10 codes and medications (ATC codes) based on consensus, grouping them into broader disease categories (Additional file 1: Tables S1 and S2). The look-back period was limited to 5 years prior to the baseline albuminuria measurement for both clinical diagnoses and dispensed medications, in order to capture active conditions. Additionally, we included a 6-month look-forward period to account for any diagnoses that may have been present at baseline but coded later, possibly due to the diagnostic cascade triggered by (1) the albuminuria test; (2) any other concurrently performed lab test; or (3) the indication of said tests. We defined multimorbidity as the presence of two or more conditions, and examined it by grouping conditions into data-driven disease patterns.

### Kidney function

Albuminuria was ascertained using all available tests and methods used in the region and performed in connection with an outpatient encounter. This included urine albumin-to-creatinine ratio (UACR), 24-h urine albumin excretion, urine protein-to-creatinine ratio (UPCR), dipstick tests and urine albumin concentration. UPCR and dipstick tests were converted to UACR values using previously defined methodology [20] and each test was categorized by the KDIGO classification (A1, < 30 mg/g; A2, 30 to 300 mg/g; A3, > 300 mg/g) [21]. eGFR was calculated using the creatinine-based revised Lund-Malmö equation [22], which is the equation automatically reported by Swedish laboratories and that has demonstrated the best agreement with measured GFR in SCREAM [23].

The primary outcome in this study was the time to the development of albuminuria grades A2 or A3, while the secondary outcome was time to the development of grade A3 albuminuria, hereafter referred to as macroalbuminuria. To confirm the presence of albuminuria, participants were required to have at least two consecutive tests above the threshold.

### Statistical analysis

#### Latent class analysis

Latent Class Analysis, a statistical modeling technique, was used to derive multimorbidity patterns. These patterns were determined separately for three age groups, with conditions having a prevalence over 2% within each age group included in the pattern derivation. The optimal number of classes was selected using the adjusted Bayesian Information Criterion and clinical interpretability of the obtained patterns. Participants were assigned to the pattern in which they had the highest probability of membership, based on the extracted posterior probabilities of membership in each of the patterns. To identify overexpressed conditions within the patterns, two measures were utilized: observed/expected (O/E) ratio and disease exclusivity. The O/E ratio was calculated by dividing the observed prevalence of a condition in the pattern by its overall prevalence, while exclusivity was determined by dividing the number of participants with a given condition in the pattern by the total number of participants with that condition. Consistent with prior research, conditions with an O/E ratio greater than two or exclusivity above 25% were considered overexpressed within the pattern. Those satisfying both criteria were used to characterize the patterns and inform the naming process. Patterns in which no condition met both criteria were labeled as “Unspecific”.

#### Association analyses

To quantify the associations between multimorbidity patterns and the incidence of albuminuria, we employed Cox regression models, using attained age as the time scale to obtain hazard ratios (HRs) with 95% confidence intervals (CIs). Participants were followed until the earliest occurrence of outcome development, emigration from Stockholm County, initiation of kidney replacement therapy, or death; otherwise, they were administratively censored on December 31, 2021. We tested the proportional hazards assumption using the Schoenfeld residual test. Additionally, to assess the cumulative incidence of the outcome while accounting for the competing risk of death, we conducted secondary analyses using Fine and Gray models to obtain subdistribution hazard ratios (sHRs) with 95% CIs. Cumulative incidence functions were estimated using the Aalen-Johansen method, accounting for death as a competing risk. Cumulative incidence of both outcomes at the median follow-up time was calculated for each multimorbidity pattern, and absolute risk differences were calculated compared to the reference group of individuals with no multimorbidity. Bootstrapping with 2000 repetitions was used to calculate 95% CIs for both the cumulative incidence estimates and their differences between patterns. All models were adjusted for age, sex, and eGFR. We also tested for multiplicative interactions between multimorbidity patterns, sex, and eGFR.

#### Sensitivity analyses

To assess the robustness of our main findings, we conducted several sensitivity analyses. First, since we used attained age as the time scale, we adjusted the models for participants’ baseline age to account for potential birth cohort effects [24]. Next, we adjusted for the baseline UACR value to evaluate the results’ sensitivity to differences in mean baseline values across the groups. Finally, to address uncertainty in the assignment to multimorbidity patterns, we reran the models, weighting individuals by their posterior probability of membership in each pattern (i.e., individuals that were more strongly assigned to the pattern contributed more information to the model).

Statistical analyses were conducted using Stata 18 (StataCorp LLC, College Station, TX) and R (R Core Team, Vienna, Austria).

## Results

### Participant characteristics

The study population included 675,570 individuals, of which 374,132 (55.4%) were female (Table 1). 470,121 (69.6%) participants were aged between 18 and 64, 120,460 (17.8%) between 65 and 74, and 84,989 (12.6%) were over the age of 75 years. The prevalence of multimorbidity and reduced eGFR (below 60 ml/min/1.73m^2^) was 72.4% and 10.6%, respectively. The five most common groups of chronic conditions were hypertension, neurotic and stress-related diseases, other musculoskeletal diseases, diabetes, and depression and mood disorders (Additional file 1: Table S3).

**Table 1:** Baseline characteristics of study sample stratified by age group

	Total	18–64 years	65–74 years	≥ 75 years
	*N* = 675,570	*N* = 470,121	*N* = 120,460	*N* = 84,989
Age	54.0 (± 17.9)	45.0 (± 13.1)	69.7 (± 2.9)	81.5 (± 4.9)
Sex (female)	55.4 (374,132)	54.9 (257,920)	52.9 (63,771)	61.7 (52,441)
Number of conditions	3.4 (± 2.8)	2.7 (± 2.3)	4.3 (± 2.8)	5.8 (± 3.3)
0–1	27.6 (186,202)	35.0 (164,634)	13.3 (16,066)	6.5 (5,502)
2–3	32.3 (218,012)	34.8 (163,522)	31.0 (37,343)	20.2 (17,147)
4–6	27.2 (183,710)	23.0 (108,308)	36.5 (43,913)	37.1 (31,489)
≥ 7	13.0 (87,646)	7.2 (33,657)	19.2 (23,138)	36.3 (30,851)
Multimorbidity	72.4 (489,368)	65.0 (305,487)	86.7 (104,394)	93.5 (79,487)
eGFR ml/min/1.73 m^2^	82 (70–93)	88 (79–98)	71 (64–78)	60 (50–68)
eGFR < 60 ml/min/1.73 m^2^	10.6 (71,758)	1.9 (8,833)	15.9 (19,200)	51.4 (43,725)

Data are presented as mean (± SD) or median (IQR) for continuous measures, and % (*n*) for categorical measures

#### *Participants aged* ≥ *75 years*

##### Multimorbidity patterns

**Fig. 1:**
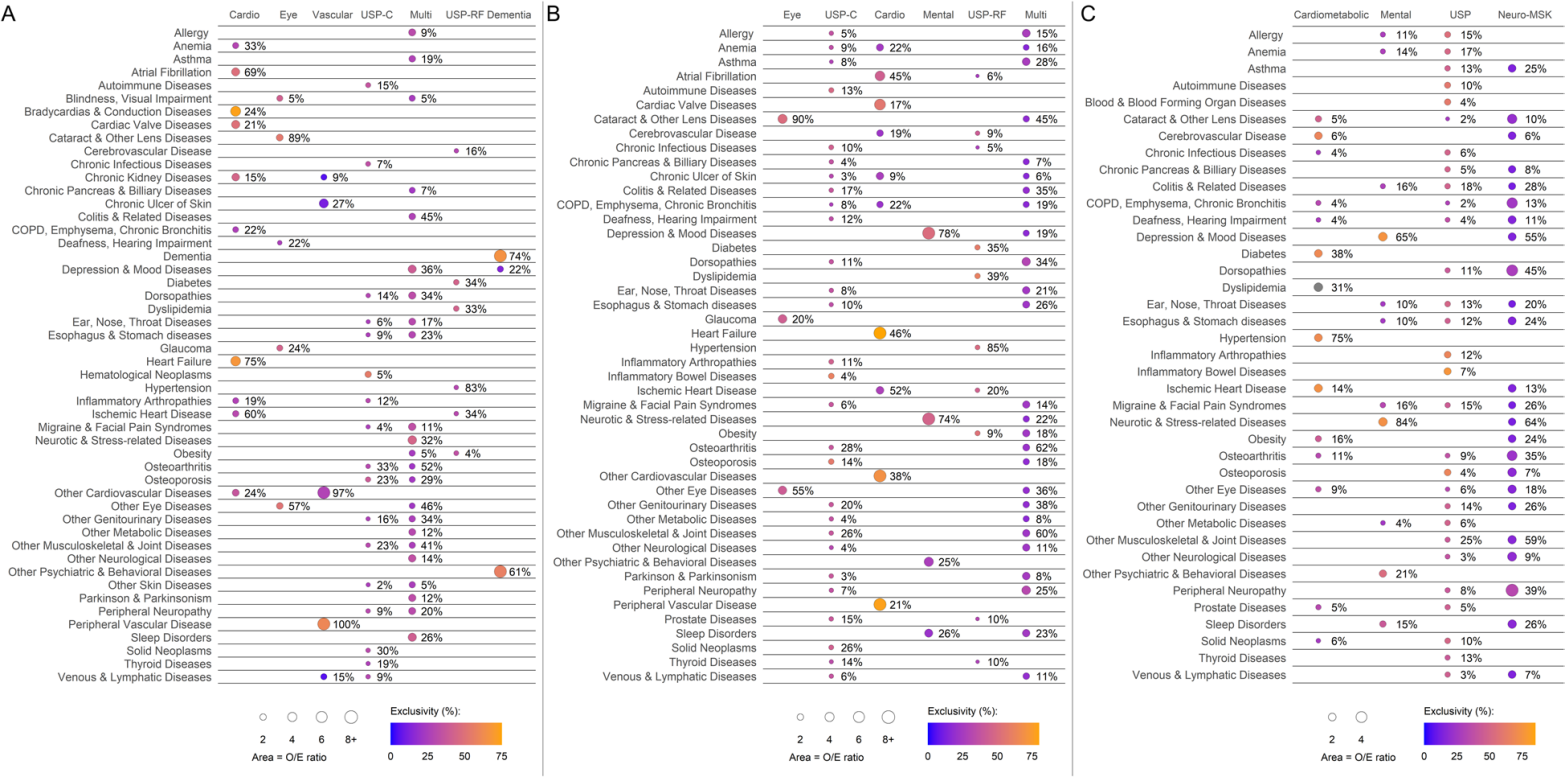
Observed/expected ratios and exclusivity values for each of the overexpressed chronic conditions among those aged ≥ 75 years (**A**), 65 to 74 years (**B**), and 18 to 64 years (**C**). Note: percentages represent the prevalence of the condition within the pattern; Abbreviations: COPD, chronic obstructive pulmonary disease; MSK, musculoskeletal; O/E, observed-to-expected; USP-C, unspecific-complex; USP-RF, unspecific-risk factors

Among 79,487 individuals with multimorbidity (93.5%), we identified seven patterns (Fig. 1, panel A). Two *Unspecific* patterns, making up 42.1%, differed in their composition—one dominated by CKD risk factors (e.g., diabetes, hypertension, obesity), and the other more heterogeneous. The remaining patterns included *Cardiovascular* (12.4%), *Eye* (18.9%), *Multisystem* (10.2%), *Dementia* (5.7%), and a *Vascular* (2.4%) pattern, the latter of which was characterized almost exclusively by peripheral and other cardiovascular diseases. A detailed description of these patterns is provided in Additional file 1: Tables S4 and S5, and the albuminuria testing rates are summarized in Additional file 1: Table S6.

##### Association analyses

In age, sex, and eGFR-adjusted Cox models, all patterns except *Dementia* were associated with a higher hazard of developing albuminuria compared to those without multimorbidity, with the *Vascular* (HR 2.41, 95% CI: 2.08–2.80) and *Cardiovascular* (HR 1.81, 95% CI: 1.62–2.02) patterns showing the strongest associations (Table 2). Associations with incident macroalbuminuria followed a similar trend.

**Table 2:** Results from Cox models exploring the association between multimorbidity and incident albuminuria

			Time to albuminuria (KDIGO A2 +)		Time to macroalbuminuria (KDIGO A3)	
Age group	Multimorbidity pattern	Group size	Number of events	Hazard ratio^a^	Number of events	Hazard ratio^a^
75 and above	*Total stratum*	84,989	8306			
	Vascular	2000 (2.4%)	281	2.41 (2.08,2.80)	68	2.10 (1.55,2.83)
	Unspecific (risk factors)	18,626 (22.0%)	2,564	1.86 (1.68,2.05)	583	1.58 (1.29,1.92)
	Cardiovascular	10,559 (12.4%)	1088	1.81 (1.62,2.02)	251	1.47 (1.18,1.84)
	Multisystem	8647 (10.2%)	581	1.45 (1.29,1.64)	127	1.30 (1.01,1.68)
	Eye	16,047 (18.9%)	1283	1.44 (1.30,1.61)	293	1.30 (1.05,1.61)
	Unspecific (complex)	18,785 (22.1%)	1820	1.43 (1.29,1.58)	485	1.50 (1.23,1.84)
	Dementia	4823 (5.7%)	224	0.94 (0.80,1.11)	57	0.95 (0.69,1.30)
	No multimorbidity	5502 (6.5%)	465	Ref	118	Ref
65 to 74	*Total stratum*	120,460	11,183		2562	
	Cardiovascular	8973 (7.5%)	1482	3.09 (2.85,3.34)	380	2.86 (2.43,3.37)
	Unspecific (risk factors)	37,297 (31.0%)	4441	2.05 (1.92,2.19)	924	1.72 (1.50,1.99)
	Multisystem	8016 (6.7%)	602	1.99 (1.80,2.20)	150	2.23 (1.81,2.73)
	Eye	11,321 (9.4%)	834	1.71 (1.57,1.88)	189	1.65 (1.37,2.00)
	Mental health	6089 (5.1%)	381	1.60 (1.42,1.80)	83	1.63 (1.27,2.09)
	Unspecific (complex)	32,698 (27.1%)	2375	1.36 (1.27,1.46)	590	1.47 (1.26,1.70)
	No multimorbidity	16,066 (13.3%)	1068	Ref	246	Ref
18 to 64	*Total stratum*	470,121	20,640		4444	
	Cardiometabolic	90,184 (19.2%)	7853	3.27 (3.14,3.41)	1644	3.08 (2.82,3.36)
	Neuro-musculoskeletal	18,645 (4.0%)	1122	3.09 (2.89,3.31)	235	3.09 (2.66,3.58)
	Unspecific	117,315 (24.9%)	5265	1.97 (1.89,2.05)	1242	2.20 (2.01,2.40)
	Mental health	79,343 (16.9%)	2345	1.63 (1.55,1.72)	469	1.58 (1.41,1.77)
	No multimorbidity	164,634 (35.0%)	4055	Ref	854	Ref

^a^ models adjusted for age (as underlying time-scale), sex, and eGFR

During follow-up, 36,520 individuals (43.0%) died without developing albuminuria, and 40,238 (47.3%) died without developing macroalbuminuria (Table 3). After accounting for the competing risk of death through Fine-Gray models, hazard estimates for albuminuria were attenuated for all patterns, with the *Dementia* pattern showing a significantly lower cumulative incidence (sHR 0.63, 95% CI: 0.47–0.88). The competing risk effect was even more pronounced for the outcome of macroalbuminuria, where the *Dementia* pattern remained inversely associated, and additionally the *Cardiovascular*, *Eye*, and *Multisystem* patterns were no longer statistically significant.

**Table 3:** Results from Fine and Gray regression models exploring the association between multimorbidity and incident albuminuria

		Time to albuminuria (KDIGO A2 +)				Time to macroalbuminuria (KDIGO A3)			
Age group	Multimorbidity pattern	Death	Cumulative incidence at median follow-up time		Subdistribution hazard ratio (sHR)^#^	Death	Cumulative incidence at median follow-up time		Subdistribution hazard ratio (sHR)^#^
			Estimate (%)	Difference (%)			Estimate (%)	Difference (%)	
75 and above	*Total stratum*	43.0%				47.3%			
	Vascular	59.6%	14.0 (12.5;15.7)	7.2 (5.5;8.8)	1.74 (1.50,2.02)	67.8%	3.1 (2.4;3.9)	1.5 (0.7;2.3)	1.52 (1.13,2.06)
	Unspecific (risk factors)	42.4%	12.1 (11.6;12.6)	5.2 (4.5;6.0)	1.69 (1.53,1.87)	48.6%	2.5 (2.2;2.7)	0.9 (0.6;1.2)	1.45 (1.19,1.77)
	Cardiovascular	62.3%	10.5 (9.9;11.2)	3.7 (2.8;4.6)	1.24 (1.11,1.39)	68.1%	2.2 (1.9;2.5)	0.6 (0.2;1.1)	1.04 (0.83,1.29)
	Multisystem	34.2%	9.3 (8.5;10.1)	2.4 (1.4;3.4)	1.17 (1.03,1.32)	36.9%	2.1 (1.8;2.5)	0.6 (0.1;1.0)	1.09 (0.85,1.40)
	Eye	28.0%	9.6 (9.1;10.2)	2.8 (2.0;3.6)	1.29 (1.16,1.44)	30.8%	2.2 (1.9;2.5)	0.6 (0.2;1.0)	1.22 (0.98,1.51)
	Unspecific (complex)	41.5%	9.4 (8.9;9.9)	2.6 (1.8;3.3)	1.30 (1.17,1.43)	45.7%	2.4 (2.1;2.6)	0.8 (0.4;1.2)	1.38 (1.13,1.69)
	Dementia	66.0%	5.5 (4.8;6.2)	− 1.3 (− 2.3; − 0.4)	0.63 (0.54,0.74)	68.5%	1.4 (1.1;1.8)	− 0.2 (− 0.6;0.3)	0.64 (0.47,0.88)
	No multimorbidity	44.1%	6.8 (6.2;7.5)	Ref	Ref	47.6%	1.6 (1.3;1.9)	Ref	Ref
65 to 74	*Total stratum*	14.8%				16.9%			
	Cardiovascular	31.3%	13.8 (13.1;14.5)	8.7 (8.0;9.4)	2.45 (2.26,2.65)	37.2%	2.5 (2.2;2.8)	1.6 (1.3;1.9)	2.28 (1.94,2.69)
	Unspecific (risk factors)	13.8%	10.1 (9.8;10.5)	5.0 (4.6;5.5)	1.96 (1.84,2.10)	16.4%	1.6 (1.4;1.7)	0.7 (0.5;0.8)	1.68 (1.46,1.94)
	Multisystem	12.2%	9.8 (9.1;10.6)	4.7 (3.9;5.5)	1.80 (1.63,1.99)	13.7%	2.1 (1.8;2.5)	1.2 (0.9;1.6)	2.11 (1.72,2.59)
	Eye	9.8%	8.7 (8.1;9.3)	3.6 (2.9;4.2)	1.63 (1.49,1.79)	11.3%	1.6 (1.4;1.9)	0.7 (0.5;1.0)	1.69 (1.40,2.05)
	Mental health	15.8%	7.7 (7.0;8.6)	2.6 (1.8;3.5)	1.39 (1.24,1.56)	17.3%	1.4 (1.2;1.8)	0.6 (0.2;0.9)	1.41 (1.10,1.81)
	Unspecific (complex)	15.1%	6.8 (6.5;7.1)	1.7 (1.3;2.1)	1.28 (1.19,1.38)	16.7%	1.3 (1.2;1.5)	0.4 (0.3;0.6)	1.40 (1.21,1.63)
	No multimorbidity	11.5%	5.1 (4.8;5.4)	Ref	Ref	12.6%	0.9 (0.8;1.0)	Ref	Ref
18 to 64	*Total stratum*	3.0%				3.3%			
	Cardiometabolic	5.5%	7.0 (6.8;7.2)	4.8 (4.6;5.0)	3.22 (3.09,3.35)	6.4%	1.2 (1.2;1.3)	0.9 (0.8;0.9)	3.29 (3.01,3.59)
	Neuro-musculoskeletal	5.6%	6.3 (5.9;6.7)	4.1 (3.7;4.4)	2.81 (2.63,3.01)	6.4%	1.2 (1.0;1.4)	0.8 (0.7;1.0)	3.09 (2.66,3.58)
	Unspecific	3.5%	4.2 (4.1;4.4)	2.0 (1.9;2.2)	1.91 (1.83,1.99)	3.8%	0.8 (0.8;0.9)	0.5 (0.4;0.5)	2.22 (2.03,2.42)
	Mental health	2.2%	3.5 (3.3;3.7)	1.3 (1.1;1.4)	1.57 (1.49,1.65)	2.3%	0.6 (0.5;0.7)	0.2 (0.2;0.3)	1.58 (1.41,1.77)
	No multimorbidity	1.4%	2.2 (2.1;2.3)	Ref	Ref	1.4%	0.4 (0.3;0.4)	Ref	Ref

All models adjusted for age, sex, and eGFR. 95% CIs in parentheses

Median follow-up time: 5 years for age group “75 and above”, 6 years for “65 to 74” and “18 to 64”

**Fig. 2:**
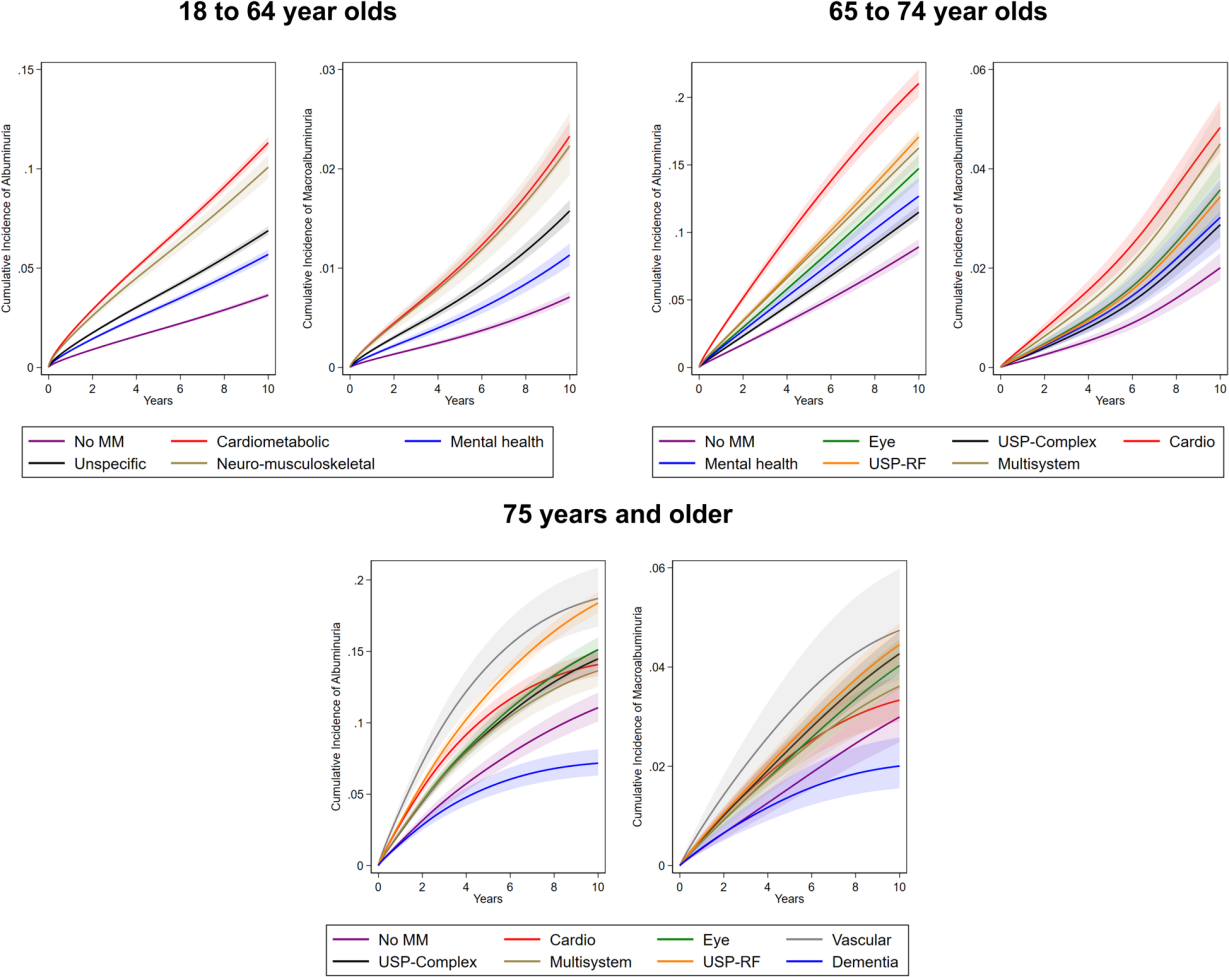
Cumulative incidence of albuminuria and macroalbuminuria over a 10-year period. Abbreviations: MM: multimorbidity; RF: risk factors; USP: unspecific. Information on numbers remaining at risk is provided in Additional file 1: Table S13

The predicted cumulative incidence for each pattern over a 10-year period is presented in Fig. 2. At the median follow-up of 5 years, the cumulative incidence of albuminuria was highest in the *Vascular* pattern (14.0%), 7.2 percentage points higher than in those without multimorbidity (6.8%). The *Cardiovascular* and *Unspecific, risk factors* patterns showed cumulative incidences of 10.5% and 12.1%, exceeding the reference by 3.7 and 5.2 percentage points, respectively. The *Dementia* pattern had a lower cumulative incidence of 5.5%, 1.3 percentage points lower than the no multimorbidity group.

##### Interaction and sensitivity analyses

We identified statistically significant interactions (Additional file 1: Fig. S2, panels A and B) between reduced eGFR and two multimorbidity patterns: *Cardiovascular* (*p* = 0.004) and *Unspecific, risk factors* (*p* = 0.026) in their association with albuminuria risk. Compared to individuals with similar kidney function and no multimorbidity, stronger associations with future albuminuria were observed among individuals without reduced kidney function (eGFR < 60 ml/min/1.73 m^2^; HR 2.21, 95% CI: 1.85–2.63 for *Cardiovascular* and HR 2.08, 95% CI: 1.80–2.40 for *Unspecific, risk factors*) compared to those with reduced kidney function (HR 1.58, 95% CI: 1.36–1.82 and HR 1.66, 95% CI: 1.45–1.91, respectively).

While similar trends were observed for other patterns, they did not reach statistical significance. No significant interactions with sex were detected. All results from the primary analyses remained robust in sensitivity analyses (Additional file 1: Tables S7 and S8).

#### Participants aged 65 to 74 years

##### Multimorbidity patterns

Among 104,394 individuals with multimorbidity (86.7%), we identified six patterns (Fig. 1, panel B). The two largest patterns, accounting for over half of participants, were *Unspecific*—one primarily linked to CKD risk factors (e.g., diabetes, hypertension, obesity) and the other encompassing a broader mix of conditions across several organ systems. The remaining patterns included *Eye* (9.4%), *Cardiovascular* (7.5%), *Multisystem* (6.7%), and *Mental Health (5.1%)*. The *Multisystem* pattern was predominantly characterized by neuro-musculoskeletal and respiratory conditions. A detailed description of these patterns is provided in Additional file 1: Tables S9 and S10, and the albuminuria testing rates are summarized in Additional file 1: Table S6.

##### Association analyses

All patterns were associated with an increased hazard of albuminuria compared to those without multimorbidity (Table 2). The strongest associations were observed for the *Cardiovascular* (HR 3.09, 95% CI: 2.85–3.34) and *Unspecific, risk factors* (HR 2.05, 95% CI: 1.92–2.19) patterns. Estimates for the secondary outcome of macroalbuminuria followed a similar pattern.

During follow-up, 17,770 individuals (14.8%) died without developing albuminuria, and 20,380 (16.9%) died without developing macroalbuminuria. After adjusting for the competing risk of death, all hazard estimates remained statistically significant, though slightly attenuated (Table 3).

The predicted cumulative incidence for each pattern over a 10-year period is presented in Fig. 2. At the median follow-up of 6 years, the *Cardiovascular* pattern showed the highest cumulative incidence of albuminuria (13.8%), 8.7 percentage points higher than the lowest (5.1%) cumulative incidence observed in those without multimorbidity. The *Unspecific, risk factors* pattern reached 10.1%, exceeding the reference by 5.0 percentage points. Other patterns ranged from 6.8% to 9.8%, corresponding to absolute differences of 1.7 to 4.7 percentage points compared to the group without multimorbidity. The predicted cumulative incidence for each pattern over a 10-year period is presented in Fig. 2.

##### Interaction and sensitivity analyses

The interaction analyses (Additional file 1: Fig. S2, panels C and D) revealed a significant interaction between the *Cardiovascular* pattern and sex (*p* = 0.006). Compared to individuals of the same sex without multimorbidity, females assigned to the Cardiovascular pattern had a 25% higher hazard of developing albuminuria than males (HR 3.60, 95% CI: 3.16–4.10 vs. HR 2.87, 95% CI: 2.60–3.16). No significant interactions were observed between multimorbidity patterns and reduced eGFR. All main results remained robust to the sensitivity analyses (Additional file 1: Tables S7 and S8).

#### Participants aged 18 to 64 years

##### Multimorbidity patterns

Among 305,487 (65.0%) individuals with multimorbidity, we identified four patterns (Fig. 1, panel C). The largest was an *Unspecific* pattern (24.9%), lacking conditions that met both overexpression criteria. The remaining three patterns were *Cardiometabolic (*19.2%), *Mental Health* (16.9%), and *Neuro-musculoskeletal* (4.0%). A detailed description of these patterns is provided in Additional file 1: Tables S11 and S12, and the albuminuria testing rates are summarized in Additional file 1: Table S6.

##### Association analyses

All patterns were associated with an increased hazard of albuminuria compared to those without multimorbidity (Table 2). The strongest associations were observed for the *Cardiovascular* (HR 3.27, 95% CI: 3.14–3.41) and *Neuro-musculoskeletal* (HR 3.09, 95% CI: 2.89–3.31) patterns. Results for the secondary outcome of macroalbuminuria were similar.

Unlike in the older age groups, sHRs from Fine-Gray models were largely similar to HRs from the Cox model, indicating that the competing risk of death had minimal influence on these estimates, consistent with the overall low proportion of deaths (3.3%) in this group (Table 3).

The predicted cumulative incidence for each pattern over a 10-year period is presented in Fig. 2. At the median follow-up of 6 years, the *Cardiometabolic* pattern showed the highest cumulative incidence of albuminuria (7.0%), 4.8 percentage points higher than the lowest cumulative incidence observed among those without multimorbidity (2.2%). The *Neuro-musculoskeletal* pattern reached 6.3%, 4.1% higher than the reference. Other patterns ranged from 3.5% to 4.2%, corresponding to absolute differences of 1.3 to 2.0 percentage points compared to the group without multimorbidity.

##### Interaction and sensitivity analyses

Statistically significant interactions were observed between multimorbidity patterns, sex, and eGFR (Additional file 1: Fig. S2, panels E and F). Compared to individuals of the same sex without multimorbidity, females assigned to the *Unspecific*, *Cardiovascular*, and *Neuro-musculoskeletal* patterns had a lower risk of albuminuria than males, whereas females in the *Mental Health* pattern had a higher hazard. There was also a significant interaction between reduced eGFR and the *Unspecific* pattern. Compared to individuals with similar kidney function and no multimorbidity, those in the *Unspecific* pattern with reduced eGFR had a higher hazard of albuminuria than those with preserved eGFR (HR 2.55, 95% CI: 2.07–3.13 vs. HR 1.90, 95% CI: 1.82–1.98).

## Discussion

In this large cohort without albuminuria at study entry, we identified patterns of multimorbidity associated with incident albuminuria, providing insight into both potential causal pathways and the absolute risk of albuminuria in real-world practice. These patterns were characterized both by conditions affecting specific organ systems (e.g., cardiovascular, mental health, eye) as well as complex combinations spanning multiple systems. We observed clear age-related trends: older age groups exhibited a greater number and broader diversity of multimorbidity patterns, including a dementia-specific one, whereas younger adults more commonly presented musculoskeletal-related multimorbidity. In addition, the interplay of sex and baseline kidney function appeared to further modify how multimorbidity patterns shaped albuminuria risk.

Our findings indicate that multimorbidity patterns are consistently associated with an increased risk of both micro- and macroalbuminuria, underscoring the heterogeneity of CKD risk pathways across the lifespan. Several patterns, including *Unspecific*, *Mental health*, *Cardiovascular*, and *Multisystem*, appeared to be associated with risks across all age groups, likely reflecting shared pathophysiological mechanisms such as chronic inflammation, endothelial dysfunction, and metabolic abnormalities known to contribute to kidney injury. The dementia-related pattern was the only one not associated with increased albuminuria risk, a result possibly attributable to differential healthcare utilization, as suggested by the lowest testing rate within this group, or healthy survivor bias. Additionally, we identified a smaller but clinically relevant vascular multimorbidity pattern among those aged ≥ 75 years, characterized by a particularly high risk of albuminuria, underscoring the direct impact of vascular diseases on kidney damage via impaired vascular integrity and renal perfusion. Patterns predominantly featuring musculoskeletal conditions in younger participants exhibited high risk of albuminuria. These risks were comparable to that of patterns characterized by traditional CKD risk factors, potentially highlighting pathways involving chronic use of nephrotoxic medications, such as NSAIDs [25], or systemic inflammatory processes. Importantly, these associations were independent of concurrently measured eGFR, indicating the potential of such patterns as clinically relevant determinants of early kidney damage, rather than reflections of existing kidney function impairment.

In competing risk analyses accounting for death, associations between multimorbidity patterns and albuminuria were attenuated in older adults, particularly affecting the cumulative incidence of macroalbuminuria among the oldest participants, with several associations becoming nonsignificant. This attenuation was particularly evident in patterns characterized by cardiovascular and multisystem diseases, despite the overexpression of several established CKD risk factors in such patterns. Cumulative incidence estimates supported these findings by showing that, although such patterns reached absolute 5-year albuminuria incidence exceeding 10–14%, their risk of progression to macroalbuminuria remained modest, and differences relative to other patterns were smaller. Thus, while these multimorbidity patterns significantly increase the likelihood of developing moderate albuminuria, the progression to severe albuminuria appears less frequent, likely because individuals with these burdened health profiles often experience other health events leading to death before kidney damage advances further. This aligns with previous findings that most older adults with CKD die of other causes before progressing to ESKD [26].

In general, females exhibited a similar or lower risk of albuminuria compared to males, particularly in younger age groups. This finding aligns with previous research highlighting the protective effects of estrogen on kidney processes, as well as the lower prevalence of cardiovascular risk factors and healthier lifestyle behaviors, such as reduced tobacco and alcohol consumption among Swedish females [27]. The increased risk observed among females with cardiovascular conditions among those aged between 65 and 74 years, one of the main exceptions, could be explained by estrogen reduction in the early postmenopause. Another notable exception was the increased risk among females assigned to the mental health pattern in individuals aged 18 to 64 years. This finding may reflect somatic symptoms being misattributed to psychological illnesses in females, a concept known as diagnostic overshadowing [28]. Given that our multimorbidity assessment relied on recorded diagnoses and medication dispensation, these females might indeed have had uncaptured conditions characteristic of the other identified patterns, all of which included CKD risk factors. An alternative explanation could be that females experience more severe or chronic mental health conditions, leading to prolonged physiological stress responses or the adoption of harmful lifestyle behaviors implicated in CKD pathogenesis (e.g., unhealthy dietary patterns, tobacco use).

We observed some interesting trends when investigating the modifying effect of reduced kidney function (i.e., eGFR < 60 ml/min/1.73 m2), although few interactions reached statistical significance. Reduced eGFR was associated with increased albuminuria risk among younger individuals, particularly those without significant multimorbidity. In contrast, such associations were attenuated or even suggested a protective trend among older individuals. These trends may reflect important distinctions between pathological and physiological declines in kidney function. Reduced eGFR in younger adults likely represents true pathological impairment, directly influencing albuminuria development [29]. Conversely, among older adults, eGFR reductions often occur due to normal aging processes and may not indicate substantial kidney pathology [29, 30], potentially diminishing the observed associations. Additionally, the creatinine-based equations used to estimate eGFR become less accurate in older populations, possibly contributing to inaccurate classification when using a fixed threshold [31]. Finally, older individuals with confirmed kidney impairment might receive intensified clinical attention, which could mitigate progression to detectable albuminuria.

Given that individuals with multimorbidity often face substantial medication burdens, our findings have important clinical implications. Initiating additional antiproteinuric medications, such as RAS inhibitors or SGLT2 inhibitors, in individuals with multimorbidity may pose challenges due to increased risk of drug interactions, adverse effects, and reduced adherence [32]. By identifying specific multimorbidity patterns associated with the early onset of albuminuria—a sensitive indicator of kidney damage that precedes kidney function decline—our study highlights opportunities for targeted screening and early intervention. The cumulative incidence estimates further reinforce these priorities, demonstrating that even within a 5- to 6-year window, several high-risk patterns experienced absolute risk differences of 5 to 9 percentage points compared to those without multimorbidity. Importantly, some of the identified patterns—such as those characterized predominantly by mental health conditions, eye disorders, or musculoskeletal and neurological diseases—include patient groups not currently prioritized for albuminuria screening in clinical guidelines. Recognizing albuminuria in these at-risk but under-recognized populations provides a window for timely preventive strategies, including lifestyle modification, optimal cardiovascular risk management, and medication review. Such proactive measures may reduce the need for additional pharmacological treatment, improve patient safety, and enhance quality of life in this large and growing segment of the population.

Our study benefits from a large sample derived from a universally accessible healthcare system, enabling comprehensive population coverage and statistical power for subgroup and interaction analyses. Additionally, our comprehensive analytical approach allowed us to examine the associations between multimorbidity and albuminuria from multiple complementary perspectives, enhancing both biological understanding and public health relevance. Nonetheless, several limitations should be acknowledged. First, reliance on healthcare utilization data may introduce selection and ascertainment bias, as individuals undergoing albuminuria testing or receiving chronic disease diagnoses are more likely to have greater healthcare contact and a higher burden of conditions that typically prompt testing, such as diabetes or hypertension. However, a large part of the population potentially missed would consist of individuals without multimorbidity—and therefore without a clinical indication for testing—which would likely strengthen, rather than attenuate, the associations observed since those without multimorbidity served as the reference group. Moreover, findings may have limited generalizability to healthcare settings with different healthcare delivery models or population demographics, although patterns identified closely resemble those reported in systematic reviews synthesizing multimorbidity patterns [3, 4], likely reflecting the Swedish publicly funded healthcare model’s extensive population coverage that enables comprehensive identification of chronic conditions. Lastly, residual confounding from factors not ascertainable in administrative data cannot be ruled out.

## Conclusions

In conclusion, distinct multimorbidity patterns, including those characterized by conditions not traditionally recognized as CKD risk factors, were associated with increased albuminuria risk in a large population undergoing albuminuria testing. Notably, we identified high-risk patterns involving discordant conditions—such as mental health disorders, eye diseases, and neuro-musculoskeletal conditions—which are not typically considered in kidney disease risk stratification. Recognizing these patterns can help clinicians identify high-risk individuals earlier and inform targeted interventions to prevent or delay kidney disease onset.

## Supplementary Information

Additional file 1: Table S1. ICD-10 codes included in each chronic disease category. Table S2. Drugs used to define chronic disease groups. Table S3. Prevalence of chronic conditions in total sample and age strata. Table S4. Conditions characterizing each multimorbidity pattern among 75 + year olds (shaded meet both overexpression criteria). Table S5. Description of patterns (75 + year olds). Table S6. Albuminuria testing rates during follow-up stratified by age group and multimorbidity pattern. Table S7. Sensitivity analyses for primary outcome (time to A2 +). Table S8. Sensitivity analyses for secondary outcome (time to A3). Table S9. Conditions characterizing each multimorbidity pattern among 65 to 74 year olds (shaded meet both overexpression criteria). Table S10. Description of patterns (65 to 74 year olds). Table S11. Conditions characterizing each multimorbidity pattern among 18–64 year olds (shaded meet both overexpression criteria). Table S12. Description of patterns (18 to 64 year olds). Table S13. Numbers remaining at risk over a 10-year period. Figure S1. Study flowchart. Figure S2. Interactions between multimorbidity patterns and sex (panels A,C,E) and eGFR (Panels B,D,F) with the outcome of albuminuria.

## Data Availability

The data contain patient-related information and cannot be shared publicly as per European General Data Protection Regulation. The data can be accessed through collaborative research applications addressed to the principal investigator JJC (juan.jesus.carrero@ki.se), and subjected to data sharing agreements that fulfil institutional and national regulations.

## Abbreviations

ATC: Anatomical Therapeutic Chemical Classification System
CI: Confidence interval
CKD: Chronic kidney disease
eGFR: Estimated glomerular filtration rate
ESKD: End-stage kidney disease
HR: Hazard ratio
ICD-10: International Classification of Diseases, 10th Revision
KDIGO: Kidney Disease: Improving Global Outcomes
NSAID: Non-steroidal anti-inflammatory drug
O/E: Observed-to-expected
RAS: Renin–angiotensin system
SCREAM: Stockholm CREAtinine Measurements project
sHR: Subdistribution hazard ratio
SGLT2: Sodium–glucose cotransporter-2 inhibitor
UACR: Urine albumin-to-creatinine ratio
UPCR: Urine protein-to-creatinine ratio
VAL: Stockholm Regional Healthcare Data Warehouse
