# Supplementary material for: Multimorbidity patterns and the subsequent risk of albuminuria: findings from the Stockholm Creatinine Measurements (SCREAM) project
Source: BMC Med. 2026 Mar 24;24:213. doi: 10.1186/s12916-026-04772-5 (PMC13063769; doi:10.1186/s12916-026-04772-5)
Supplement: Supplementary file 1 — Additional file 1: Table S1. ICD-10 codes included in each chronic disease category. Table S2. Drugs used to define chronic disease groups. Table S3. Prevalence of chronic conditions in total sample and age strata. Table S4. Conditions characterizing each multimorbidity pattern among 75 + year olds (shaded meet both overexpression criteria). Table S5. Description of patterns (75 + year olds). Table S6. Albuminuria testing rates during follow-up stratified by age group and multimorbidity pattern. Table S7. Sensitivity analyses for primary outcome (time to A2 +). Table S8. Sensitivity analyses for secondary outcome (time to A3). Table S9. Conditions characterizing each multimorbidity pattern among 65 to 74 year olds (shaded meet both overexpression criteria). Table S10. Description of patterns (65 to 74 year olds). Table S11. Conditions characterizing each multimorbidity pattern among 18–64 year olds (shaded meet both overexpression criteria). Table S12. Description of patterns (18 to 64 year olds). Table S13. Numbers remaining at risk over a 10-year period. Figure S1. Study flowchart. Figure S2. Interactions between multimorbidity patterns and sex (panels A,C,E) and eGFR (Panels B,D,F) with the outcome of albuminuria. [file 12916_2026_4772_MOESM1_ESM.docx]

**Table S1.** ICD-10 codes included in each chronic disease category.

| **ALLERGY** | | | |
| --- | --- | --- | --- |
| **Included ICD-10 codes and labels** | | | |
| J301 | Allergic rhinitis due to pollen | | |
| J302 | Other seasonal allergic rhinitis | | |
| J303 | Other allergic rhinitis | | |
| J304 | Allergic rhinitis, unspecified | | |
| J450 | Predominantly allergic asthma | | |
| K522 | Allergic and dietetic gastroenteritis and colitis | | |
| L20 | Atopic dermatitis | | |
| L23 | Allergic contact dermatitis | | |
| L500 | Allergic urticaria | | |
| Z516 | Desensitization to allergens | | |
| **ANEMIA** | | | |
| **Included ICD-10 codes and labels** | | | |
| D50 | Iron deficiency anaemia | | |
| D51 | Vitamin B12 deficiency anaemia | | |
| D52 | Folate deficiency anaemia | | |
| D53 | Other nutritional anaemias | | |
| D55 | Anaemia due to enzyme disorders | | |
| D560 | | Alpha thalassaemia | |
| D561 | | Beta thalassaemia | |
| D562 | | Delta-beta thalassaemia | |
| D564 | | Hereditary persistence of fetal haemoglobin [HPFH] | |
| D568 | | Other thalassaemias | |
| D569 | | Thalassaemia, unspecified | |
| D57 | Sickle-cell disorders | | |
| D58 | Other hereditary haemolytic anaemias | | |
| D591 | | Other autoimmune haemolytic anaemias | |
| D594 | | Other nonautoimmune haemolytic anaemias | |
| D595 | | Paroxysmal nocturnal haemoglobinuria [Marchiafava-Micheli] | |
| D598 | | Other acquired haemolytic anaemias | |
| D599 | | Acquired haemolytic anaemia, unspecified | |
| D600 | | Chronic acquired pure red cell aplasia | |
| D608 | | Other acquired pure red cell aplasias | |
| D609 | | Acquired pure red cell aplasia, unspecified | |
| D610 | | Constitutional aplastic anaemia | |
| D613 | | Idiopathic aplastic anaemia | |
| D618 | | Other specified aplastic anaemias | |
| D619 | | Aplastic anaemia, unspecified | |
| D63 | Anaemia in chronic diseases classified elsewhere | | |
| D640 | | Hereditary sideroblastic anaemia | |
| D641 | | Secondary sideroblastic anaemia due to disease | |
| D643 | | Other sideroblastic anaemias | |
| D644 | | Congenital dyserythropoietic anaemia | |
| D648 | | Other specified anaemias | |
| D649 | | Anaemia, unspecified | |
| **ASTHMA** | | | |
| **Included ICD-10 codes and labels** | | | |
| J45 | Asthma | | |
| **ATRIAL FIBRILLATION** | | | |
| **Included ICD-10 codes and labels** | | | |
| I48 | Atrial fibrillation and flutter | | |
| **AUTOIMMUNE DISEASES** | | | |
| **Included ICD-10 codes and labels** | | | |
| I731 | Thromboangiitis obliterans [Buerger] | | |
| L100 | Pemphigus vulgaris | | |
| L101 | Pemphigus vegetans | | |
| L102 | Pemphigus foliaceus | | |
| L103 | Brazilian pemphigus [fogo selvagem] | | |
| L104 | Pemphigus erythematosus | | |
| L108 | Other pemphigus | | |
| L109 | Pemphigus, unspecified | | |
| L12 | Pemphigoid | | |
| L40 | Psoriasis | | |
| L41 | Parapsoriasis | | |
| L93 | Lupus erythematosus | | |
| L94 | Other localized connective tissue disorders | | |
| L95 | Vasculitis limited to skin, not elsewhere classified | | |
| M30 | Polyarteritis nodosa and related conditions | | |
| M31 | Other necrotizing vasculopathies | | |
| M321 | | | Systemic lupus erythematosus with organ or system involvement |
| M328 | | | Other forms of systemic lupus erythematosus |
| M329 | | | Systemic lupus erythematosus, unspecified |
| M33 | Dermatopolymyositis | | |
| M340 | | | Progressive systemic sclerosis |
| M341 | | | CR(E)ST syndrome |
| M348 | | | Other forms of systemic sclerosis |
| M349 | | | Systemic sclerosis, unspecified |
| M350 | | | Sicca syndrome [Sjögren] |
| M351 | | | Other overlap syndromes |
| M352 | | | Behçet disease |
| M353 | | | Polymyalgia rheumatica |
| M354 | | | Diffuse (eosinophilic) fasciitis |
| M355 | | | Multifocal fibrosclerosis |
| M356 | | | Relapsing panniculitis [Weber-Christian] |
| M364 | | | Arthropathy in hypersensitivity reactions classified elsewhere |
| M368 | | | Systemic disorders of connective tissue in other diseases classified elsewhere |
| **BLINDNESS, VISUAL IMPAIRMENT** | | | |
| **Included ICD-10 codes and labels** | | | |
| H540 | | Blindness, binocular | |
| H541 | | Severe visual impairment, binocular | |
| H542 | | Moderate visual impairment, binocular | |
| H544 | | Blindness, monocular | |
| H545 | | Severe visual impairment, monocular | |
| H546 | | Moderate visual impairment, monocular | |
| H549 | | Unspecified visual impairment (binocular) | |
| Z442 | Fitting and adjustment of artificial eye | | |
| Z970 | Presence of artificial eye | | |
| **BLOOD AND BLOOD FORMING ORGAN DISEASES** | | | |
| **Included ICD-10 codes and labels** | | | |
| D66 | Hereditary factor VIII deficiency | | |
| D67 | Hereditary factor IX deficiency | | |
| D680 | | Von Willebrand disease | |
| D681 | | Hereditary factor XI deficiency | |
| D682 | | Hereditary deficiency of other clotting factors | |
| D685 | | Primary thrombophilia | |
| D686 | | Other thrombophilia | |
| D688 | | Other specified coagulation defects | |
| D689 | | Coagulation defect, unspecified | |
| D690 | | Allergic purpura | |
| D691 | | Qualitative platelet defects | |
| D692 | | Other nonthrombocytopenic purpura | |
| D693 | | Idiopathic thrombocytopenic purpura | |
| D694 | | Other primary thrombocytopenia | |
| D696 | | Thrombocytopenia, unspecified | |
| D698 | | Other specified haemorrhagic conditions | |
| D699 | | Haemorrhagic condition, unspecified | |
| D71 | Functional disorders of polymorphonuclear neutrophils | | |
| D720 | Genetic anomalies of leukocytes | | |
| D730 | Hyposplenism | | |
| D731 | Hypersplenism | | |
| D732 | Chronic congestive splenomegaly | | |
| D740 | | Congenital methaemoglobinaemia | |
| D749 | | Methaemoglobinaemia, unspecified | |
| D750 | Familial erythrocytosis | | |
| D761 | Haemophagocytic lymphohistiocytosis | | |
| D763 | Other histiocytosis syndromes | | |
| D77 | Other disorders of blood and blood-forming organs in diseases classified elsewhere | | |
| D800 | | Hereditary hypogammaglobulinaemia | |
| D801 | | Nonfamilial hypogammaglobulinaemia | |
| D802 | | Selective deficiency of immunoglobulin A [IgA] | |
| D803 | | Selective deficiency of immunoglobulin G [IgG] subclasses | |
| D804 | | Selective deficiency of immunoglobulin M [IgM] | |
| D805 | | Immunodeficiency with increased immunoglobulin M [IgM] | |
| D806 | | Antibody deficiency with near-normal immunoglobulins or with hyperimmunoglobulinaemia | |
| D808 | | Other immunodeficiencies with predominantly antibody defects | |
| D809 | | Immunodeficiency with predominantly antibody defects, unspecified | |
| D81 | Combined immunodeficiencies | | |
| D82 | Immunodeficiency associated with other major defects | | |
| D83 | Common variable immunodeficiency | | |
| D84 | Other immunodeficiencies | | |
| D86 | Sarcoidosis | | |
| D890 | | Polyclonal hypergammaglobulinaemia | |
| D892 | | Hypergammaglobulinaemia, unspecified | |
| D898 | | Other specified disorders involving the immune mechanism, not elsewhere classified | |
| D899 | | Disorder involving the immune mechanism, unspecified | |
| **BRADYCARDIAS AND CONDUCTION DISEASES** | | | |
| **Included ICD-10 codes and labels** | | | |
| I441 | Atrioventricular block, second degree | | |
| I442 | Atrioventricular block, complete | | |
| I443 | Other and unspecified atrioventricular block | | |
| I453 | Trifascicular block | | |
| I455 | Other specified heart block | | |
| Z950 | Presence of cardiac pacemaker | | |
| **CARDIAC VALVE DISEASES** | | | |
| **Included ICD-10 codes and labels** | | | |
| I05 | Rheumatic mitral valve diseases | | |
| I06 | Rheumatic aortic valve diseases | | |
| I07 | Rheumatic tricuspid valve diseases | | |
| I08 | Multiple valve diseases | | |
| I091 | Rheumatic diseases of endocardium, valve unspecified | | |
| I098 | Other specified rheumatic heart diseases | | |
| I34 | Nonrheumatic mitral valve disorders | | |
| I35 | Nonrheumatic aortic valve disorders | | |
| I36 | Nonrheumatic tricuspid valve disorders | | |
| I37 | Pulmonary valve disorders | | |
| I38 | Endocarditis, valve unspecified | | |
| I390 | Mitral valve disorders in diseases classified elsewhere | | |
| I391 | Aortic valve disorders in diseases classified elsewhere | | |
| I392 | Tricuspid valve disorders in diseases classified elsewhere | | |
| I393 | Pulmonary valve disorders in diseases classified elsewhere | | |
| I394 | Multiple valve disorders in diseases classified elsewhere | | |
| Q22 | Congenital malformations of pulmonary and tricuspid valves | | |
| Q23 | Congenital malformations of aortic and mitral valves | | |
| Z952 | Presence of prosthetic heart valve | | |
| Z953 | Presence of xenogenic heart valve | | |
| Z954 | Presence of other heart-valve replacement | | |
| **CATARACT AND OTHER LENS DISEASES** | | | |
| **Included ICD-10 codes and labels** | | | |
| H25 | Senile cataract | | |
| H26 | Other cataract | | |
| H27 | Other disorders of lens | | |
| H28 | Cataract and other disorders of lens in diseases classified elsewhere | | |
| Q12 | Congenital lens malformations | | |
| Z961 | Presence of intraocular lens | | |
| **CEREBROVASCULAR DISEASE** | | | |
| **Included ICD-10 codes and labels** | | | |
| G45 | Transient cerebral ischaemic attacks and related syndromes | | |
| G46 | Vascular syndromes of brain in cerebrovascular diseases | | |
| I60 | Subarachnoid haemorrhage | | |
| I61 | Intracerebral haemorrhage | | |
| I62 | Other nontraumatic intracranial haemorrhage | | |
| I63 | Cerebral infarction | | |
| I64 | Stroke, not specified as haemorrhage or infarction | | |
| I67 | Other cerebrovascular diseases | | |
| I69 | Sequelae of cerebrovascular disease | | |
| **CHROMOSOMAL ABNORMALITIES** | | | |
| **Included ICD-10 codes and labels** | | | |
| Q90 | Down syndrome | | |
| Q91 | Edwards syndrome and Patau syndrome | | |
| Q92 | Other trisomies and partial trisomies of the autosomes, not elsewhere classified | | |
| Q93 | Monosomies and deletions from the autosomes, not elsewhere classified | | |
| Q95 | Balanced rearrangements and structural markers, not elsewhere classified | | |
| Q96 | Turner syndrome | | |
| Q97 | Other sex chromosome abnormalities, female phenotype, not elsewhere classified | | |
| Q98 | Other sex chromosome abnormalities, male phenotype, not elsewhere classified | | |
| Q99 | Other chromosome abnormalities, not elsewhere classified | | |
| **CHRONIC INFECTIOUS DISEASES** | | | |
| **Included ICD-10 codes and labels** | | | |
| A15 | Respiratory tuberculosis, bacteriologically and histologically confirmed | | |
| A16 | Respiratory tuberculosis, not confirmed bacteriologically or histologically | | |
| A17 | Tuberculosis of nervous system | | |
| A18 | Tuberculosis of other organs | | |
| A19 | Miliary tuberculosis | | |
| A30 | Leprosy [Hansen disease] | | |
| A31 | Infection due to other mycobacteria | | |
| A50 | Congenital syphilis | | |
| A52 | Late syphilis | | |
| A53 | Other and unspecified syphilis | | |
| A65 | Nonvenereal syphilis | | |
| A66 | Yaws | | |
| A67 | Pinta [carate] | | |
| A692 | Lyme disease | | |
| A81 | Atypical virus infections of central nervous system | | |
| B20 | Human immunodeficiency virus [HIV] disease resulting in infectious and parasitic diseases | | |
| B21 | Human immunodeficiency virus [HIV] disease resulting in malignant neoplasms | | |
| B22 | Human immunodeficiency virus [HIV] disease resulting in other specified diseases | | |
| B23 | Human immunodeficiency virus [HIV] disease resulting in other conditions | | |
| B24 | Unspecified human immunodeficiency virus [HIV] disease | | |
| B381 | Chronic pulmonary coccidioidomycosis | | |
| B391 | Chronic pulmonary histoplasmosis capsulati | | |
| B401 | Chronic pulmonary blastomycosis | | |
| B572 | Chagas disease (chronic) with heart involvement | | |
| B573 | Chagas disease (chronic) with digestive system involvement | | |
| B574 | Chagas disease (chronic) with nervous system involvement | | |
| B575 | Chagas disease (chronic) with other organ involvement | | |
| B65 | Schistosomiasis [bilharziasis] | | |
| B92 | Sequelae of leprosy | | |
| B94 | Sequelae of other and unspecified infectious and parasitic diseases | | |
| J65 | Pneumoconiosis associated with tuberculosis | | |
| M863 | Chronic multifocal osteomyelitis | | |
| M864 | Chronic osteomyelitis with draining sinus | | |
| M865 | Other chronic haematogenous osteomyelitis | | |
| M866 | Other chronic osteomyelitis | | |
| **CHRONIC KIDNEY DISEASES** | | | |
| **Included ICD-10 codes and labels** | | | |
| I120 | Hypertensive renal disease with renal failure | | |
| I130 | Hypertensive heart and renal disease with (congestive) heart failure | | |
| I131 | Hypertensive heart and renal disease with renal failure | | |
| I132 | Hypertensive heart and renal disease with both (congestive) heart failure and renal failure | | |
| I139 | Hypertensive heart and renal disease, unspecified | | |
| N01 | Rapidly progressive nephritic syndrome | | |
| N03 | Chronic nephritic syndrome | | |
| N04 | Nephrotic syndrome | | |
| N05 | Unspecified nephritic syndrome | | |
| N07 | Hereditary nephropathy, not elsewhere classified | | |
| N08 | Glomerular disorders in diseases classified elsewhere | | |
| N11 | Chronic tubulo-interstitial nephritis | | |
| N183 | Chronic kidney disease, stage 3 | | |
| N184 | Chronic kidney disease, stage 4 | | |
| N185 | Chronic kidney disease, stage 5 | | |
| N189 | Chronic kidney disease, unspecified | | |
| Q60 | Renal agenesis and other reduction defects of kidney | | |
| Q611 | Polycystic kidney, autosomal recessive | | |
| Q612 | Polycystic kidney, autosomal dominant | | |
| Q613 | Polycystic kidney, unspecified | | |
| Q614 | Renal dysplasia | | |
| Q615 | Medullary cystic kidney | | |
| Q618 | Other cystic kidney diseases | | |
| Q619 | Cystic kidney disease, unspecified | | |
| Z905 | Acquired absence of kidney | | |
| Z940 | Kidney transplant status | | |
| **CHRONIC LIVER DISEASES** | | | |
| **Included ICD-10 codes and labels** | | | |
| B18 | Chronic viral hepatitis | | |
| K702 | | Alcoholic fibrosis and sclerosis of liver | |
| K703 | | Alcoholic cirrhosis of liver | |
| K704 | | Alcoholic hepatic failure | |
| K709 | | Alcoholic liver disease, unspecified | |
| K713 | Toxic liver disease with chronic persistent hepatitis | | |
| K714 | Toxic liver disease with chronic lobular hepatitis | | |
| K715 | Toxic liver disease with chronic active hepatitis | | |
| K717 | Toxic liver disease with fibrosis and cirrhosis of liver | | |
| K721 | Chronic hepatic failure | | |
| K73 | Chronic hepatitis, not elsewhere classified | | |
| K74 | Fibrosis and cirrhosis of liver | | |
| K753 | Granulomatous hepatitis, not elsewhere classified | | |
| K754 | Autoimmune hepatitis | | |
| K758 | Other specified inflammatory liver diseases | | |
| K761 | Chronic passive congestion of liver | | |
| K766 | Portal hypertension | | |
| K767 | Hepatorenal syndrome | | |
| K778 | Liver disorders in other diseases classified elsewhere | | |
| Q446 | Cystic disease of liver | | |
| Z944 | Liver transplant status | | |
| **CHRONIC PANCREAS, BILIARY TRACT AND GALLBLADDER DISEASES** | | | |
| **Included ICD-10 codes and labels** | | | |
| K800 | Calculus of gallbladder with acute cholecystitis | | |
| K801 | Calculus of gallbladder with other cholecystitis | | |
| K802 | Calculus of gallbladder without cholecystitis | | |
| K808 | Other cholelithiasis | | |
| K811 | Chronic cholecystitis | | |
| K860 | | Alcohol-induced chronic pancreatitis | |
| K861 | | Other chronic pancreatitis | |
| K868 | | Other specified diseases of pancreas | |
| Q440 | Agenesis, aplasia and hypoplasia of gallbladder | | |
| Q441 | Other congenital malformations of gallbladder | | |
| Q442 | Atresia of bile ducts | | |
| Q443 | Congenital stenosis and stricture of bile ducts | | |
| Q444 | Choledochal cyst | | |
| Q445 | Other congenital malformations of bile ducts | | |
| Q450 | Agenesis, aplasia and hypoplasia of pancreas | | |
| **CHRONIC ULCER OF THE SKIN** | | | |
| **Included ICD-10 codes and labels** | | | |
| I830 | Varicose veins of lower extremities with ulcer | | |
| I832 | Varicose veins of lower extremities with both ulcer and inflammation | | |
| L89 | Decubitus ulcer and pressure area | | |
| L97 | Ulcer of lower limb, not elsewhere classified | | |
| L984 | Chronic ulcer of skin, not elsewhere classified | | |
| **COLITIS AND RELATED DISEASES** | | | |
| **Included ICD-10 codes and labels** | | | |
| K520 | Gastroenteritis and colitis due to radiation | | |
| K528 | Other specified noninfective gastroenteritis and colitis | | |
| K551 | Chronic vascular disorders of intestine | | |
| K552 | Angiodysplasia of colon | | |
| K572 | Diverticular disease of large intestine with perforation and abscess | | |
| K573 | Diverticular disease of large intestine without perforation or abscess | | |
| K574 | Diverticular disease of both small and large intestine with perforation and abscess | | |
| K575 | Diverticular disease of both small and large intestine without perforation or abscess | | |
| K578 | Diverticular disease of intestine, part unspecified, with perforation and abscess | | |
| K579 | Diverticular disease of intestine, part unspecified, without perforation or abscess | | |
| K58 | Irritable bowel syndrome | | |
| K590 | Constipation | | |
| K592 | Neurogenic bowel, not elsewhere classified | | |
| K622 | | Anal prolapse | |
| K623 | | Rectal prolapse | |
| K624 | | Stenosis of anus and rectum | |
| K627 | | Radiation proctitis | |
| K628 | | Other specified diseases of anus and rectum | |
| K629 | | Disease of anus and rectum, unspecified | |
| K634 | Enteroptosis | | |
| K640 | | First degree haemorrhoids | |
| K641 | | Second degree haemorrhoids | |
| K642 | | Third degree haemorrhoids | |
| K643 | | Fourth degree haemorrhoids | |
| K644 | | Residual haemorrhoidal skin tags | |
| K648 | | Other specified haemorrhoids | |
| K649 | | Haemorrhoids, unspecified | |
| **COPD, EMPHYSEMA, CHRONIC BRONCHITIS** | | | |
| **Included ICD-10 codes and labels** | | | |
| J41 | Simple and mucopurulent chronic bronchitis | | |
| J42 | Unspecified chronic bronchitis | | |
| J43 | Emphysema | | |
| J44 | Other chronic obstructive pulmonary disease | | |
| J47 | Bronchiectasis | | |
| **DEAFNESS, HEARING IMPAIRMENT** | | | |
| **Included ICD-10 codes and labels** | | | |
| H80 | Otosclerosis | | |
| H90 | Conductive and sensorineural hearing loss | | |
| H911 | Presbycusis | | |
| H913 | Deaf mutism, not elsewhere classified | | |
| H919 | Hearing loss, unspecified | | |
| Q16 | Congenital malformations of ear causing impairment of hearing | | |
| Z453 | Adjustment and management of implanted hearing device | | |
| Z461 | Fitting and adjustment of hearing aid | | |
| Z962 | Presence of otological and audiological implants | | |
| Z974 | Presence of external hearing-aid | | |
| **DEMENTIA** | | | |
| **Included ICD-10 codes and labels** | | | |
| F00 | Dementia in Alzheimer disease | | |
| F01 | Vascular dementia | | |
| F02 | Dementia in other diseases classified elsewhere | | |
| F03 | Unspecified dementia | | |
| F051 | Delirium superimposed on dementia | | |
| G30 | Alzheimer disease | | |
| G31 | Other degenerative diseases of nervous system, not elsewhere classified | | |
| **DEPRESSION AND MOOD DISEASES** | | | |
| **Included ICD-10 codes and labels** | | | |
| F30 | Manic episode | | |
| F31 | Bipolar affective disorder | | |
| F32 | Depressive episode | | |
| F33 | Recurrent depressive disorder | | |
| F34 | Persistent mood [affective] disorders | | |
| F38 | Other mood [affective] disorders | | |
| F39 | Unspecified mood [affective] disorder | | |
| F412 | Mixed anxiety and depressive disorder | | |
| **DIABETES** | | | |
| **Included ICD-10 codes and labels** | | | |
| E10 | Insulin-dependent diabetes mellitus | | |
| E11 | Non-insulin-dependent diabetes mellitus | | |
| E13 | Other specified diabetes mellitus | | |
| E14 | Unspecified diabetes mellitus | | |
| E891 | Postprocedural hypoinsulinaemia | | |
| **DORSOPATHIES** | | | |
| **Included ICD-10 codes and labels** | | | |
| M40 | Kyphosis and lordosis | | |
| M41 | Scoliosis | | |
| M42 | Spinal osteochondrosis | | |
| M43 | Other deforming dorsopathies | | |
| M47 | Spondylosis | | |
| M48 | Other spondylopathies | | |
| M49 | Spondylopathies in diseases classified elsewhere | | |
| M50 | Cervical disc disorders | | |
| M51 | Other intervertebral disc disorders | | |
| M53 | Other dorsopathies, not elsewhere classified | | |
| Q675 | Congenital deformity of spine | | |
| Q761 | Klippel-Feil syndrome | | |
| Q764 | Other congenital malformations of spine, not associated with scoliosis | | |
| **DYSLIPIDEMIA** | | | |
| **Included ICD-10 codes and labels** | | | |
| E78 | Disorders of lipoprotein metabolism and other lipidaemias | | |
| **EAR, NOSE, THROAT DISEASES** | | | |
| **Included ICD-10 codes and labels** | | | |
| H604 | Cholesteatoma of external ear | | |
| H661 | Chronic tubotympanic suppurative otitis media | | |
| H662 | Chronic atticoantral suppurative otitis media | | |
| H663 | Other chronic suppurative otitis media | | |
| H701 | Chronic mastoiditis | | |
| H71 | Cholesteatoma of middle ear | | |
| H731 | Chronic myringitis | | |
| H741 | Adhesive middle ear disease | | |
| H810 | MÚniÞre disease | | |
| H831 | Labyrinthine fistula | | |
| H832 | Labyrinthine dysfunction | | |
| H95 | Postprocedural disorders of ear and mastoid process, not elsewhere classified | | |
| J300 | Vasomotor rhinitis | | |
| J31 | Chronic rhinitis, nasopharyngitis and pharyngitis | | |
| J32 | Chronic sinusitis | | |
| J33 | Nasal polyp | | |
| J341 | Cyst and mucocele of nose and nasal sinus | | |
| J342 | Deviated nasal septum | | |
| J343 | Hypertrophy of nasal turbinates | | |
| J35 | Chronic diseases of tonsils and adenoids | | |
| J37 | Chronic laryngitis and laryngotracheitis | | |
| J380 | Paralysis of vocal cords and larynx | | |
| J386 | Stenosis of larynx | | |
| K051 | Chronic gingivitis | | |
| K053 | Chronic periodontitis | | |
| K07 | Dentofacial anomalies [including malocclusion] | | |
| K110 | Atrophy of salivary gland | | |
| K117 | Disturbances of salivary secretion | | |
| Q30 | Congenital malformations of nose | | |
| Q31 | Congenital malformations of larynx | | |
| Q32 | Congenital malformations of trachea and bronchus | | |
| Q35 | Cleft palate | | |
| Q36 | Cleft lip | | |
| Q37 | Cleft palate with cleft lip | | |
| Q38 | Other congenital malformations of tongue, mouth and pharynx | | |
| **EPILEPSY** | | | |
| **Included ICD-10 codes and labels** | | | |
| G400 | | Localization-related (focal)(partial) idiopathic epilepsy and epileptic syndromes with seizures of localized onset | |
| G401 | | Localization-related (focal)(partial) symptomatic epilepsy and epileptic syndromes with simple partial seizures | |
| G402 | | Localization-related (focal)(partial) symptomatic epilepsy and epileptic syndromes with complex partial seizures | |
| G403 | | Generalized idiopathic epilepsy and epileptic syndromes | |
| G404 | | Other generalized epilepsy and epileptic syndromes | |
| G406 | | Grand mal seizures, unspecified (with or without petit mal) | |
| G407 | | Petit mal, unspecified, without grand mal seizures | |
| G408 | | Other epilepsy | |
| G409 | | Epilepsy, unspecified | |
| **ESOPHAGUS, STOMACH AND DUODENUM DISEASES** | | | |
| **Included ICD-10 codes and labels** | | | |
| I85 | Oesophageal varices | | |
| I864 | Gastric varices | | |
| I982 | Oesophageal varices without bleeding in diseases classified elsewhere | | |
| I983 | Oesophageal varices with bleeding in diseases classified elsewhere | | |
| K21 | Gastro-oesophageal reflux disease | | |
| K220 | Achalasia of cardia | | |
| K222 | Oesophageal obstruction | | |
| K224 | Dyskinesia of oesophagus | | |
| K225 | Diverticulum of oesophagus, acquired | | |
| K227 | Barrett oesophagus | | |
| K230 | Tuberculous oesophagitis | | |
| K231 | Megaoesophagus in Chagas disease | | |
| K254 | Gastric ulcer: Chronic or unspecified with haemorrhage | | |
| K255 | Gastric ulcer: Chronic or unspecified with perforation | | |
| K256 | Gastric ulcer: Chronic or unspecified with both haemorrhage and perforation | | |
| K257 | Gastric ulcer: Chronic without haemorrhage or perforation | | |
| K264 | Duodenal ulcer: Chronic or unspecified with haemorrhage | | |
| K265 | Duodenal ulcer: Chronic or unspecified with perforation | | |
| K266 | Duodenal ulcer: Chronic or unspecified with both haemorrhage and perforation | | |
| K267 | Duodenal ulcer: Chronic without haemorrhage or perforation | | |
| K274 | Peptic ulcer, site unspecified: Chronic or unspecified with haemorrhage | | |
| K275 | Peptic ulcer, site unspecified: Chronic or unspecified with perforation | | |
| K276 | Peptic ulcer, site unspecified: Chronic or unspecified with both haemorrhage and perforation | | |
| K277 | Peptic ulcer, site unspecified: Chronic without haemorrhage or perforation | | |
| K284 | Gastrojejunal ulcer: Chronic or unspecified with haemorrhage | | |
| K285 | Gastrojejunal ulcer: Chronic or unspecified with perforation | | |
| K286 | Gastrojejunal ulcer: Chronic or unspecified with both haemorrhage and perforation | | |
| K287 | Gastrojejunal ulcer: Chronic without haemorrhage or perforation | | |
| K293 | Chronic superficial gastritis | | |
| K294 | Chronic atrophic gastritis | | |
| K295 | Chronic gastritis, unspecified | | |
| K296 | Other gastritis | | |
| K297 | Gastritis, unspecified | | |
| K298 | Duodenitis | | |
| K299 | Gastroduodenitis, unspecified | | |
| K311 | Adult hypertrophic pyloric stenosis | | |
| K312 | Hourglass stricture and stenosis of stomach | | |
| K313 | Pylorospasm, not elsewhere classified | | |
| K314 | Gastric diverticulum | | |
| K315 | Obstruction of duodenum | | |
| Q39 | Congenital malformations of oesophagus | | |
| Q40 | Other congenital malformations of upper alimentary tract | | |
| Z903 | Acquired absence of part of stomach | | |
| **GLAUCOMA** | | | |
| **Included ICD-10 codes and labels** | | | |
| H401 | Primary open-angle glaucoma | | |
| H402 | Primary angle-closure glaucoma | | |
| H403 | Glaucoma secondary to eye trauma | | |
| H404 | Glaucoma secondary to eye inflammation | | |
| H405 | Glaucoma secondary to other eye disorders | | |
| H406 | Glaucoma secondary to drugs | | |
| H408 | Other glaucoma | | |
| H409 | Glaucoma, unspecified | | |
| **HEART FAILURE** | | | |
| **Included ICD-10 codes and labels** | | | |
| I110 | Hypertensive heart disease with (congestive) heart failure | | |
| I130 | Hypertensive heart and renal disease with (congestive) heart failure | | |
| I132 | Hypertensive heart and renal disease with both (congestive) heart failure and renal failure | | |
| I27 | Other pulmonary heart diseases | | |
| I280 | Arteriovenous fistula of pulmonary vessels | | |
| I42 | Cardiomyopathy | | |
| I43 | Cardiomyopathy in diseases classified elsewhere | | |
| I50 | Heart failure | | |
| I515 | Myocardial degeneration | | |
| I517 | Cardiomegaly | | |
| I528 | Other heart disorders in other diseases classified elsewhere | | |
| Z941 | Heart transplant status | | |
| Z943 | Heart and lungs transplant status | | |
| **HEMATOLOGICAL NEOPLASMS** | | | |
| **Included ICD-10 codes and labels** | | | |
| C81 | Hodgkin lymphoma | | |
| C82 | Follicular lymphoma | | |
| C83 | Non-follicular lymphoma | | |
| C84 | Mature T/NK-cell lymphomas | | |
| C85 | Other and unspecified types of non-Hodgkin lymphoma | | |
| C86 | Other specified types of T/NK-cell lymphoma | | |
| C88 | Malignant immunoproliferative diseases | | |
| C90 | Multiple myeloma and malignant plasma cell neoplasms | | |
| C91 | Lymphoid leukaemia | | |
| C92 | Myeloid leukaemia | | |
| C93 | Monocytic leukaemia | | |
| C94 | Other leukaemias of specified cell type | | |
| C95 | Leukaemia of unspecified cell type | | |
| C96 | Other and unspecified malignant neoplasms of lymphoid, haematopoietic and related tissue | | |
| **HYPERTENSION** | | | |
| **Included ICD-10 codes and labels** | | | |
| I10 | Essential (primary) hypertension | | |
| I11 | Hypertensive heart disease | | |
| I12 | Hypertensive renal disease | | |
| I13 | Hypertensive heart and renal disease | | |
| I15 | Secondary hypertension | | |
| **INFLAMMATORY ARTHROPATHIES** | | | |
| **Included ICD-10 codes and labels** | | | |
| M023 | Reiter disease | | |
| M05 | Seropositive rheumatoid arthritis | | |
| M06 | Other rheumatoid arthritis | | |
| M07 | Psoriatic and enteropathic arthropathies | | |
| M08 | Juvenile arthritis | | |
| M09 | Juvenile arthritis in diseases classified elsewhere | | |
| M10 | Gout | | |
| M11 | Other crystal arthropathies | | |
| M12 | Other specific arthropathies | | |
| M13 | Other arthritis | | |
| M14 | Arthropathies in other diseases classified elsewhere | | |
| M45 | Ankylosing spondylitis | | |
| M460 | Spinal enthesopathy | | |
| M461 | Sacroiliitis, not elsewhere classified | | |
| M468 | Other specified inflammatory spondylopathies | | |
| M469 | Inflammatory spondylopathy, unspecified | | |
| **INFLAMMATORY BOWEL DISEASES** | | | |
| **Included ICD-10 codes and labels** | | | |
| K50 | Crohn disease [regional enteritis] | | |
| K51 | Ulcerative colitis | | |
| **ISCHEMIC HEART DISEASE** | | | |
| **Included ICD-10 codes and labels** | | | |
| I20 | Angina pectoris | | |
| I21 | Acute myocardial infarction | | |
| I22 | Subsequent myocardial infarction | | |
| I24 | Other acute ischaemic heart diseases | | |
| I25 | Chronic ischaemic heart disease | | |
| Z951 | Presence of aortocoronary bypass graft | | |
| Z955 | Presence of coronary angioplasty implant and graft | | |
| **MIGRAINE AND FACIAL PAIN SYNDROMES** | | | |
| **Included ICD-10 codes and labels** | | | |
| G43 | Migraine | | |
| G440 | Cluster headache syndrome | | |
| G441 | Vascular headache, not elsewhere classified | | |
| G442 | Tension-type headache | | |
| G443 | Chronic post-traumatic headache | | |
| G448 | Other specified headache syndromes | | |
| G50 | Disorders of trigeminal nerve | | |
| **MULTIPLE SCLEROSIS** | | | |
| **Included ICD-10 codes and labels** | | | |
| G35 | Multiple sclerosis | | |
| **NEUROTIC, STRESS-RELATED AND SOMATOFORM DISEASES** | | | |
| **Included ICD-10 codes and labels** | | | |
| F40 | Phobic anxiety disorders | | |
| F41 | Other anxiety disorders | | |
| F42 | Obsessive-compulsive disorder | | |
| F431 | Post-traumatic stress disorder | | |
| F438 | Other reactions to severe stress | | |
| F439 | Reaction to severe stress, unspecified | | |
| F44 | Dissociative [conversion] disorders | | |
| F45 | Somatoform disorders | | |
| F48 | Other neurotic disorders | | |
| **OBESITY** | | | |
| **Included ICD-10 codes and labels** | | | |
| E66 | Obesity | | |
| **OSTEOARTHRITIS AND OTHER DEGENERATIVE JOINT DISEASES** | | | |
| **Included ICD-10 codes and labels** | | | |
| M15 | Polyarthrosis | | |
| M16 | Coxarthrosis [arthrosis of hip] | | |
| M17 | Gonarthrosis [arthrosis of knee] | | |
| M18 | Arthrosis of first carpometacarpal joint | | |
| M19 | Other arthrosis | | |
| M362 | Haemophilic arthropathy | | |
| M363 | Arthropathy in other blood disorders | | |
| **OSTEOPOROSIS** | | | |
| **Included ICD-10 codes and labels** | | | |
| M80 | Osteoporosis with pathological fracture | | |
| M81 | Osteoporosis without pathological fracture | | |
| M82 | Osteoporosis in diseases classified elsewhere | | |
| **OTHER CARDIOVASCULAR DISEASES** | | | |
| **Included ICD-10 codes and labels** | | | |
| I090 | Rheumatic myocarditis | | |
| I092 | Chronic rheumatic pericarditis | | |
| I099 | Rheumatic heart disease, unspecified | | |
| I281 | Aneurysm of pulmonary artery | | |
| I310 | Chronic adhesive pericarditis | | |
| I311 | Chronic constrictive pericarditis | | |
| I456 | Pre-excitation syndrome | | |
| I495 | Sick sinus syndrome | | |
| I498 | Other specified cardiac arrhythmias | | |
| I700 | Atherosclerosis of aorta | | |
| I701 | Atherosclerosis of renal artery | | |
| I708 | Atherosclerosis of other arteries | | |
| I709 | Generalized and unspecified atherosclerosis | | |
| I71 | Aortic aneurysm and dissection | | |
| I72 | Other aneurysm and dissection | | |
| I790 | Aneurysm of aorta in diseases classified elsewhere | | |
| I791 | Aortitis in diseases classified elsewhere | | |
| I950 | Idiopathic hypotension | | |
| I951 | Orthostatic hypotension | | |
| I958 | Other hypotension | | |
| Q20 | Congenital malformations of cardiac chambers and connections | | |
| Q21 | Congenital malformations of cardiac septa | | |
| Q24 | Other congenital malformations of heart | | |
| Q25 | Congenital malformations of great arteries | | |
| Q26 | Congenital malformations of great veins | | |
| Q27 | Other congenital malformations of peripheral vascular system | | |
| Q28 | Other congenital malformations of circulatory system | | |
| Z958 | Presence of other cardiac and vascular implants and grafts | | |
| Z959 | Presence of cardiac and vascular implant and graft, unspecified | | |
| **OTHER DIGESTIVE DISEASES** | | | |
| **Included ICD-10 codes and labels** | | | |
| K660 | Peritoneal adhesions | | |
| K900 | Coeliac disease | | |
| K901 | Tropical sprue | | |
| K902 | Blind loop syndrome, not elsewhere classified | | |
| K911 | Postgastric surgery syndromes | | |
| K93 | Disorders of other digestive organs in diseases classified elsewhere | | |
| Q41 | Congenital absence, atresia and stenosis of small intestine | | |
| Q42 | Congenital absence, atresia and stenosis of large intestine | | |
| Q43 | Other congenital malformations of intestine | | |
| R15 | Faecal incontinence | | |
| Z904 | Acquired absence of other parts of digestive tract | | |
| Z980 | Intestinal bypass and anastomosis status | | |
| **OTHER EYE DISEASES** | | | |
| **Included ICD-10 codes and labels** | | | |
| H022 | Lagophthalmos | | |
| H023 | Blepharochalasis | | |
| H024 | Ptosis of eyelid | | |
| H025 | Other disorders affecting eyelid function | | |
| H040 | | Dacryoadenitis | |
| H041 | | Other disorders of lacrimal gland | |
| H042 | | Epiphora | |
| H044 | | Chronic inflammation of lacrimal passages | |
| H045 | | Stenosis and insufficiency of lacrimal passages | |
| H046 | | Other changes in lacrimal passages | |
| H048 | | Other disorders of lacrimal system | |
| H049 | | Disorder of lacrimal system, unspecified | |
| H051 | | Chronic inflammatory disorders of orbit | |
| H052 | | Exophthalmic conditions | |
| H053 | | Deformity of orbit | |
| H054 | | Enophthalmos | |
| H055 | | Retained (old) foreign body following penetrating wound of orbit | |
| H058 | | Other disorders of orbit | |
| H059 | | Disorder of orbit, unspecified | |
| H104 | Chronic conjunctivitis | | |
| H17 | Corneal scars and opacities | | |
| H184 | Corneal degeneration | | |
| H185 | Hereditary corneal dystrophies | | |
| H186 | Keratoconus | | |
| H187 | Other corneal deformities | | |
| H188 | Other specified disorders of cornea | | |
| H189 | Disorder of cornea, unspecified | | |
| H193 | Keratitis and keratoconjunctivitis in other diseases classified elsewhere | | |
| H198 | Other disorders of sclera and cornea in diseases classified elsewhere | | |
| H201 | Chronic iridocyclitis | | |
| H21 | Other disorders of iris and ciliary body | | |
| H310 | Chorioretinal scars | | |
| H311 | Choroidal degeneration | | |
| H312 | Hereditary choroidal dystrophy | | |
| H318 | Other specified disorders of choroid | | |
| H319 | Disorder of choroid, unspecified | | |
| H33 | Retinal detachments and breaks | | |
| H352 | Other proliferative retinopathy | | |
| H353 | Degeneration of macula and posterior pole | | |
| H354 | Peripheral retinal degeneration | | |
| H355 | Hereditary retinal dystrophy | | |
| H357 | Separation of retinal layers | | |
| H358 | Other specified retinal disorders | | |
| H359 | Retinal disorder, unspecified | | |
| H36 | Retinal disorders in diseases classified elsewhere | | |
| H472 | | Optic atrophy | |
| H473 | | Other disorders of optic disc | |
| H474 | | Disorders of optic chiasm | |
| H475 | | Disorders of other visual pathways | |
| H476 | | Disorders of visual cortex | |
| H477 | | Disorder of visual pathways, unspecified | |
| H480 | | Optic atrophy in diseases classified elsewhere | |
| H488 | | Other disorders of optic nerve and visual pathways in diseases classified elsewhere | |
| H49 | Paralytic strabismus | | |
| H51 | Other disorders of binocular movement | | |
| Q10 | Congenital malformations of eyelid, lacrimal apparatus and orbit | | |
| Q11 | Anophthalmos, microphthalmos and macrophthalmos | | |
| Q13 | Congenital malformations of anterior segment of eye | | |
| Q14 | Congenital malformations of posterior segment of eye | | |
| Q15 | Other congenital malformations of eye | | |
| Z947 | Corneal transplant status | | |
| **OTHER GENITOURINARY DISEASES** | | | |
| **Included ICD-10 codes and labels** | | | |
| B901 | Sequelae of genitourinary tuberculosis | | |
| N200 | Calculus of kidney | | |
| N202 | Calculus of kidney with calculus of ureter | | |
| N209 | Urinary calculus, unspecified | | |
| N210 | Calculus in bladder | | |
| N218 | Other lower urinary tract calculus | | |
| N219 | Calculus of lower urinary tract, unspecified | | |
| N22 | Calculus of urinary tract in diseases classified elsewhere | | |
| N301 | Interstitial cystitis (chronic) | | |
| N302 | Other chronic cystitis | | |
| N303 | Trigonitis | | |
| N304 | Irradiation cystitis | | |
| N31 | Neuromuscular dysfunction of bladder, not elsewhere classified | | |
| N320 | Bladder-neck obstruction | | |
| N323 | Diverticulum of bladder | | |
| N328 | Other specified disorders of bladder | | |
| N329 | Bladder disorder, unspecified | | |
| N33 | Bladder disorders in diseases classified elsewhere | | |
| N35 | Urethral stricture | | |
| N393 | Stress incontinence | | |
| N394 | Other specified urinary incontinence | | |
| N480 | Leukoplakia of penis | | |
| N484 | Impotence of organic origin | | |
| N489 | Disorder of penis, unspecified | | |
| N701 | Chronic salpingitis and oophoritis | | |
| N711 | Chronic inflammatory disease of uterus | | |
| N731 | Chronic parametritis and pelvic cellulitis | | |
| N734 | Female chronic pelvic peritonitis | | |
| N736 | Female pelvic peritoneal adhesions | | |
| N761 | Subacute and chronic vaginitis | | |
| N763 | Subacute and chronic vulvitis | | |
| N81 | Female genital prolapse | | |
| N88 | Other noninflammatory disorders of cervix uteri | | |
| N895 | Stricture and atresia of vagina | | |
| N905 | Atrophy of vulva | | |
| N952 | Postmenopausal atrophic vaginitis | | |
| Q54 | Hypospadias | | |
| Q620 | Congenital hydronephrosis | | |
| Q621 | Atresia and stenosis of ureter | | |
| Q622 | Congenital megaloureter | | |
| Q623 | Other obstructive defects of renal pelvis and ureter | | |
| Q624 | Agenesis of ureter | | |
| Q627 | Congenital vesico-uretero-renal reflux | | |
| Q628 | Other congenital malformations of ureter | | |
| Q638 | Other specified congenital malformations of kidney | | |
| Q639 | Congenital malformation of kidney, unspecified | | |
| Q640 | Epispadias | | |
| Q641 | Exstrophy of urinary bladder | | |
| Q643 | Other atresia and stenosis of urethra and bladder neck | | |
| Q644 | Malformation of urachus | | |
| Q645 | Congenital absence of bladder and urethra | | |
| Q646 | Congenital diverticulum of bladder | | |
| Q647 | Other congenital malformations of bladder and urethra | | |
| Q648 | Other specified congenital malformations of urinary system | | |
| Q649 | Congenital malformation of urinary system, unspecified | | |
| Z906 | Acquired absence of other organs of urinary tract | | |
| Z907 | Acquired absence of genital organ(s) | | |
| Z960 | Presence of urogenital implants | | |
| **OTHER METABOLIC DISEASES** | | | |
| **Included ICD-10 codes and labels** | | | |
| E20 | Hypoparathyroidism | | |
| E21 | Hyperparathyroidism and other disorders of parathyroid gland | | |
| E22 | Hyperfunction of pituitary gland | | |
| E230 | Hypopituitarism | | |
| E232 | Diabetes insipidus | | |
| E233 | Hypothalamic dysfunction, not elsewhere classified | | |
| E236 | Other disorders of pituitary gland | | |
| E237 | Disorder of pituitary gland, unspecified | | |
| E240 | Pituitary-dependent Cushing disease | | |
| E241 | Nelson syndrome | | |
| E243 | Ectopic ACTH syndrome | | |
| E248 | Other Cushing syndrome | | |
| E249 | Cushing syndrome, unspecified | | |
| E25 | Adrenogenital disorders | | |
| E26 | Hyperaldosteronism | | |
| E270 | Other adrenocortical overactivity | | |
| E271 | Primary adrenocortical insufficiency | | |
| E272 | Addisonian crisis | | |
| E274 | Other and unspecified adrenocortical insufficiency | | |
| E275 | Adrenomedullary hyperfunction | | |
| E278 | Other specified disorders of adrenal gland | | |
| E279 | Disorder of adrenal gland, unspecified | | |
| E28 | Ovarian dysfunction | | |
| E29 | Testicular dysfunction | | |
| E31 | Polyglandular dysfunction | | |
| E340 | Carcinoid syndrome | | |
| E341 | Other hypersecretion of intestinal hormones | | |
| E342 | Ectopic hormone secretion, not elsewhere classified | | |
| E345 | Androgen resistance syndrome | | |
| E348 | Other specified endocrine disorders | | |
| E349 | Endocrine disorder, unspecified | | |
| E351 | Disorders of adrenal glands in diseases classified elsewhere | | |
| E358 | Disorders of other endocrine glands in diseases classified elsewhere | | |
| E40 | Kwashiorkor | | |
| E41 | Nutritional marasmus | | |
| E42 | Marasmic kwashiorkor | | |
| E43 | Unspecified severe protein-energy malnutrition | | |
| E440 | Moderate protein-energy malnutrition | | |
| E45 | Retarded development following protein-energy malnutrition | | |
| E46 | Unspecified protein-energy malnutrition | | |
| E64 | Sequelae of malnutrition and other nutritional deficiencies | | |
| E70 | Disorders of aromatic amino-acid metabolism | | |
| E71 | Disorders of branched-chain amino-acid metabolism and fatty-acid metabolism | | |
| E72 | Other disorders of amino-acid metabolism | | |
| E74 | Other disorders of carbohydrate metabolism | | |
| E75 | Disorders of sphingolipid metabolism and other lipid storage disorders | | |
| E76 | Disorders of glycosaminoglycan metabolism | | |
| E77 | Disorders of glycoprotein metabolism | | |
| E791 | Lesch-Nyhan syndrome | | |
| E798 | Other disorders of purine and pyrimidine metabolism | | |
| E799 | Disorder of purine and pyrimidine metabolism, unspecified | | |
| E800 | Hereditary erythropoietic porphyria | | |
| E801 | Porphyria cutanea tarda | | |
| E802 | Other porphyria | | |
| E803 | Defects of catalase and peroxidase | | |
| E805 | Crigler-Najjar syndrome | | |
| E806 | Other disorders of bilirubin metabolism | | |
| E807 | Disorder of bilirubin metabolism, unspecified | | |
| E83 | Disorders of mineral metabolism | | |
| E84 | Cystic fibrosis | | |
| E85 | Amyloidosis | | |
| E880 | Disorders of plasma-protein metabolism, not elsewhere classified | | |
| E881 | Lipodystrophy, not elsewhere classified | | |
| E882 | Lipomatosis, not elsewhere classified | | |
| E888 | Other specified metabolic disorders | | |
| E889 | Metabolic disorder, unspecified | | |
| E891 | Postprocedural hypoinsulinaemia | | |
| E893 | Postprocedural hypopituitarism | | |
| E894 | Postprocedural ovarian failure | | |
| E895 | Postprocedural testicular hypofunction | | |
| E896 | Postprocedural adrenocortical(-medullary) hypofunction | | |
| E898 | Other postprocedural endocrine and metabolic disorders | | |
| E899 | Postprocedural endocrine and metabolic disorder, unspecified | | |
| K903 | Pancreatic steatorrhoea | | |
| K904 | Malabsorption due to intolerance, not elsewhere classified | | |
| K908 | Other intestinal malabsorption | | |
| K909 | Intestinal malabsorption, unspecified | | |
| K912 | Postsurgical malabsorption, not elsewhere classified | | |
| M83 | Adult osteomalacia | | |
| M88 | Paget disease of bone [osteitis deformans] | | |
| N25 | Disorders resulting from impaired renal tubular function | | |
| **OTHER MUSCULOSKELETAL AND JOINT DISEASES** | | | |
| **Included ICD-10 codes and labels** | | | |
| B902 | Sequelae of tuberculosis of bones and joints | | |
| M212 | Flexion deformity | | |
| M213 | Wrist or foot drop (acquired) | | |
| M214 | Flat foot [pes planus] (acquired) | | |
| M215 | Acquired clawhand, clubhand, clawfoot and clubfoot | | |
| M216 | Other acquired deformities of ankle and foot | | |
| M217 | Unequal limb length (acquired) | | |
| M218 | Other specified acquired deformities of limbs | | |
| M219 | Acquired deformity of limb, unspecified | | |
| M22 | Disorders of patella | | |
| M23 | Internal derangement of knee | | |
| M24 | Other specific joint derangements | | |
| M252 | Flail joint | | |
| M253 | Other instability of joint | | |
| M357 | Hypermobility syndrome | | |
| M61 | Calcification and ossification of muscle | | |
| M652 | Calcific tendinitis | | |
| M653 | Trigger finger | | |
| M654 | Radial styloid tenosynovitis [de Quervain] | | |
| M700 | Chronic crepitant synovitis of hand and wrist | | |
| M720 | Palmar fascial fibromatosis [Dupuytren] | | |
| M722 | Plantar fascial fibromatosis | | |
| M724 | Pseudosarcomatous fibromatosis | | |
| M750 | Adhesive capsulitis of shoulder | | |
| M751 | Rotator cuff syndrome | | |
| M753 | Calcific tendinitis of shoulder | | |
| M754 | Impingement syndrome of shoulder | | |
| M797 | Fibromyalgia | | |
| M841 | Nonunion of fracture [pseudarthrosis] | | |
| M89 | Other disorders of bone | | |
| M91 | Juvenile osteochondrosis of hip and pelvis | | |
| M93 | Other osteochondropathies | | |
| M94 | Other disorders of cartilage | | |
| M96 | Postprocedural musculoskeletal disorders, not elsewhere classified | | |
| M99 | Biomechanical lesions, not elsewhere classified | | |
| Q65 | Congenital deformities of hip | | |
| Q66 | Congenital deformities of feet | | |
| Q68 | Other congenital musculoskeletal deformities | | |
| Q71 | Reduction defects of upper limb | | |
| Q72 | Reduction defects of lower limb | | |
| Q73 | Reduction defects of unspecified limb | | |
| Q74 | Other congenital malformations of limb(s) | | |
| Q77 | Osteochondrodysplasia with defects of growth of tubular bones and spine | | |
| Q78 | Other osteochondrodysplasias | | |
| Q796 | Ehlers-Danlos syndrome | | |
| Q798 | Other congenital malformations of musculoskeletal system | | |
| Q87 | Other specified congenital malformation syndromes affecting multiple systems | | |
| S382 | Traumatic amputation of external genital organs | | |
| S48 | Traumatic amputation of shoulder and upper arm | | |
| S58 | Traumatic amputation of forearm | | |
| S68 | Traumatic amputation of wrist and hand | | |
| S78 | Traumatic amputation of hip and thigh | | |
| S88 | Traumatic amputation of lower leg | | |
| S98 | Traumatic amputation of ankle and foot | | |
| T05 | Traumatic amputations involving multiple body regions | | |
| T096 | Traumatic amputation of trunk, level unspecified | | |
| T116 | Traumatic amputation of upper limb, level unspecified | | |
| T136 | Traumatic amputation of lower limb, level unspecified | | |
| T147 | Crushing injury and traumatic amputation of unspecified body region | | |
| T90 | Sequelae of injuries of head | | |
| T91 | Sequelae of injuries of neck and trunk | | |
| T92 | Sequelae of injuries of upper limb | | |
| T93 | Sequelae of injuries of lower limb | | |
| T94 | Sequelae of injuries involving multiple and unspecified body regions | | |
| T95 | Sequelae of burns, corrosions and frostbite | | |
| T96 | Sequelae of poisoning by drugs, medicaments and biological substances | | |
| T97 | Sequelae of toxic effects of substances chiefly nonmedicinal as to source | | |
| T98 | Sequelae of other and unspecified effects of external causes | | |
| Z440 | Fitting and adjustment of artificial arm (complete)(partial) | | |
| Z441 | Fitting and adjustment of artificial leg (complete)(partial) | | |
| Z891 | Acquired absence of hand and wrist | | |
| Z892 | Acquired absence of upper limb above wrist | | |
| Z893 | Acquired absence of both upper limbs [any level] | | |
| Z894 | Acquired absence of foot and ankle | | |
| Z895 | Acquired absence of leg at or below knee | | |
| Z896 | Acquired absence of leg above knee | | |
| Z897 | Acquired absence of both lower limbs [any level, except toes alone] | | |
| Z898 | Acquired absence of upper and lower limbs [any level] | | |
| Z899 | Acquired absence of limb, unspecified | | |
| Z946 | Bone transplant status | | |
| Z966 | Presence of orthopaedic joint implants | | |
| Z971 | Presence of artificial limb (complete)(partial) | | |
| **OTHER NEUROLOGICAL DISEASES** | | | |
| **Included ICD-10 codes and labels** | | | |
| B900 | Sequelae of central nervous system tuberculosis | | |
| D482 | Neoplasm of uncertain or unknown behaviour: Peripheral nerves and autonomic nervous system | | |
| G041 | Tropical spastic paraplegia | | |
| G09 | Sequelae of inflammatory diseases of central nervous system | | |
| G10 | Huntington disease | | |
| G11 | Hereditary ataxia | | |
| G12 | Spinal muscular atrophy and related syndromes | | |
| G132 | | Systemic atrophy primarily affecting central nervous system in myxoedema | |
| G138 | | Systemic atrophy primarily affecting central nervous system in other diseases classified elsewhere | |
| G24 | Dystonia | | |
| G250 | | Essential tremor | |
| G252 | | Other specified forms of tremor | |
| G253 | | Myoclonus | |
| G255 | | Other chorea | |
| G258 | | Other specified extrapyramidal and movement disorders | |
| G259 | | Extrapyramidal and movement disorder, unspecified | |
| G26 | Extrapyramidal and movement disorders in diseases classified elsewhere | | |
| G32 | Other degenerative disorders of nervous system in diseases classified elsewhere | | |
| G37 | Other demyelinating diseases of central nervous system | | |
| G511 | | Geniculate ganglionitis | |
| G512 | | Melkersson syndrome | |
| G513 | | Clonic hemifacial spasm | |
| G514 | | Facial myokymia | |
| G518 | | Other disorders of facial nerve | |
| G519 | | Disorder of facial nerve, unspecified | |
| G52 | Disorders of other cranial nerves | | |
| G53 | Cranial nerve disorders in diseases classified elsewhere | | |
| G70 | Myasthenia gravis and other myoneural disorders | | |
| G71 | Primary disorders of muscles | | |
| G723 | Periodic paralysis | | |
| G724 | Inflammatory myopathy, not elsewhere classified | | |
| G728 | Other specified myopathies | | |
| G729 | Myopathy, unspecified | | |
| G730 | | Myasthenic syndromes in endocrine diseases | |
| G731 | | Lambert-Eaton syndrome | |
| G735 | | Myopathy in endocrine diseases | |
| G736 | | Myopathy in metabolic diseases | |
| G737 | | Myopathy in other diseases classified elsewhere | |
| G80 | Cerebral palsy | | |
| G81 | Hemiplegia | | |
| G82 | Paraplegia and tetraplegia | | |
| G830 | | Diplegia of upper limbs | |
| G831 | | Monoplegia of lower limb | |
| G832 | | Monoplegia of upper limb | |
| G833 | | Monoplegia, unspecified | |
| G834 | | Cauda equina syndrome | |
| G835 | | Locked-in syndrome | |
| G839 | | Paralytic syndrome, unspecified | |
| G90 | Disorders of autonomic nervous system | | |
| G91 | Hydrocephalus | | |
| G938 | Other specified disorders of brain | | |
| G939 | Disorder of brain, unspecified | | |
| G95 | Other diseases of spinal cord | | |
| G99 | Other disorders of nervous system in diseases classified elsewhere | | |
| M471 | Other spondylosis with myelopathy | | |
| Q00 | Anencephaly and similar malformations | | |
| Q01 | Encephalocele | | |
| Q02 | Microcephaly | | |
| Q03 | Congenital hydrocephalus | | |
| Q04 | Other congenital malformations of brain | | |
| Q05 | Spina bifida | | |
| Q06 | Other congenital malformations of spinal cord | | |
| Q07 | Other congenital malformations of nervous system | | |
| Q760 | Spina bifida occulta | | |
| **OTHER PSYCHIATRIC AND BEHAVIORAL DISEASES** | | | |
| **Included ICD-10 codes and labels** | | | |
| F04 | Organic amnesic syndrome, not induced by alcohol and other psychoactive substances | | |
| F06 | Other mental disorders due to brain damage and dysfunction and to physical disease | | |
| F07 | Personality and behavioural disorders due to brain disease, damage and dysfunction | | |
| F09 | Unspecified organic or symptomatic mental disorder | | |
| F102 | Mental and behavioural disorders due to use of alcohol: Dependence syndrome | | |
| F106 | Mental and behavioural disorders due to use of alcohol: Amnesic syndrome | | |
| F107 | Mental and behavioural disorders due to use of alcohol: Residual and late-onset psychotic disorder | | |
| F112 | Mental and behavioural disorders due to use of opioids: Dependence syndrome | | |
| F116 | Mental and behavioural disorders due to use of opioids: Amnesic syndrome | | |
| F117 | Mental and behavioural disorders due to use of opioids: Residual and late-onset psychotic disorder | | |
| F122 | Mental and behavioural disorders due to use of cannabinoids: Dependence syndrome | | |
| F126 | Mental and behavioural disorders due to use of cannabinoids: Amnesic syndrome | | |
| F127 | Mental and behavioural disorders due to use of cannabinoids: Residual and late-onset psychotic disorder | | |
| F132 | Mental and behavioural disorders due to use of sedatives or hypnotics: Dependence syndrome | | |
| F136 | Mental and behavioural disorders due to use of sedatives or hypnotics: Amnesic syndrome | | |
| F137 | Mental and behavioural disorders due to use of sedatives or hypnotics: Residual and late-onset psychotic disorder | | |
| F142 | Mental and behavioural disorders due to use of cocaine: Dependence syndrome | | |
| F146 | Mental and behavioural disorders due to use of cocaine: Amnesic syndrome | | |
| F147 | Mental and behavioural disorders due to use of cocaine: Residual and late-onset psychotic disorder | | |
| F152 | Mental and behavioural disorders due to use of other stimulants, including caffeine: Dependence syndrome | | |
| F156 | Mental and behavioural disorders due to use of other stimulants, including caffeine: Amnesic syndrome | | |
| F157 | Mental and behavioural disorders due to use of other stimulants, including caffeine: Residual and late-onset psychotic disorder | | |
| F162 | Mental and behavioural disorders due to use of hallucinogens: Dependence syndrome | | |
| F166 | Mental and behavioural disorders due to use of hallucinogens: Amnesic syndrome | | |
| F167 | Mental and behavioural disorders due to use of hallucinogens: Residual and late-onset psychotic disorder | | |
| F172 | Mental and behavioural disorders due to use of tobacco: Dependence syndrome | | |
| F176 | Mental and behavioural disorders due to use of tobacco: Amnesic syndrome | | |
| F177 | Mental and behavioural disorders due to use of tobacco: Residual and late-onset psychotic disorder | | |
| F182 | Mental and behavioural disorders due to use of volatile solvents: Dependence syndrome | | |
| F186 | Mental and behavioural disorders due to use of volatile solvents: Amnesic syndrome | | |
| F187 | Mental and behavioural disorders due to use of volatile solvents: Residual and late-onset psychotic disorder | | |
| F192 | Mental and behavioural disorders due to multiple drug use and use of other psychoactive substances: Dependence syndrome | | |
| F196 | Mental and behavioural disorders due to multiple drug use and use of other psychoactive substances: Amnesic syndrome | | |
| F197 | Mental and behavioural disorders due to multiple drug use and use of other psychoactive substances: Residual and late-onset psychotic disorder | | |
| F50 | Eating disorders | | |
| F52 | Sexual dysfunction, not caused by organic disorder or disease | | |
| F60 | Specific personality disorders | | |
| F61 | Mixed and other personality disorders | | |
| F62 | Enduring personality changes, not attributable to brain damage and disease | | |
| F63 | Habit and impulse disorders | | |
| F68 | Other disorders of adult personality and behaviour | | |
| F70 | Mild mental retardation | | |
| F71 | Moderate mental retardation | | |
| F72 | Severe mental retardation | | |
| F73 | Profound mental retardation | | |
| F78 | Other mental retardation | | |
| F79 | Unspecified mental retardation | | |
| F80 | Specific developmental disorders of speech and language | | |
| F81 | Specific developmental disorders of scholastic skills | | |
| F82 | Specific developmental disorder of motor function | | |
| F83 | Mixed specific developmental disorders | | |
| F84 | Pervasive developmental disorders | | |
| F88 | Other disorders of psychological development | | |
| F89 | Unspecified disorder of psychological development | | |
| F95 | Tic disorders | | |
| F99 | Mental disorder, not otherwise specified | | |
| **OTHER RESPIRATORY DISEASES** | | | |
| **Included ICD-10 codes and labels** | | | |
| B909 | Sequelae of respiratory and unspecified tuberculosis | | |
| E662 | Extreme obesity with alveolar hypoventilation | | |
| J60 | Coalworker pneumoconiosis | | |
| J61 | Pneumoconiosis due to asbestos and other mineral fibres | | |
| J62 | Pneumoconiosis due to dust containing silica | | |
| J63 | Pneumoconiosis due to other inorganic dusts | | |
| J64 | Unspecified pneumoconiosis | | |
| J65 | Pneumoconiosis associated with tuberculosis | | |
| J66 | Airway disease due to specific organic dust | | |
| J67 | Hypersensitivity pneumonitis due to organic dust | | |
| J684 | Chronic respiratory conditions due to chemicals, gases, fumes and vapours | | |
| J701 | Chronic and other pulmonary manifestations due to radiation | | |
| J703 | Chronic drug-induced interstitial lung disorders | | |
| J704 | Drug-induced interstitial lung disorders, unspecified | | |
| J84 | Other interstitial pulmonary diseases | | |
| J92 | Pleural plaque | | |
| J941 | Fibrothorax | | |
| J953 | Chronic pulmonary insufficiency following surgery | | |
| J955 | Postprocedural subglottic stenosis | | |
| J961 | Chronic respiratory failure | | |
| J980 | Diseases of bronchus, not elsewhere classified | | |
| J982 | Interstitial emphysema | | |
| J983 | Compensatory emphysema | | |
| J984 | Other disorders of lung | | |
| J985 | Diseases of mediastinum, not elsewhere classified | | |
| J986 | Disorders of diaphragm | | |
| J988 | Other specified respiratory disorders | | |
| J989 | Respiratory disorder, unspecified | | |
| Q33 | Congenital malformations of lung | | |
| Q34 | Other congenital malformations of respiratory system | | |
| Z902 | Acquired absence of lung [part of] | | |
| Z942 | Lung transplant status | | |
| Z943 | Heart and lungs transplant status | | |
| Z963 | Presence of artificial larynx | | |
| **OTHER SKIN DISEASES** | | | |
| **Included ICD-10 codes and labels** | | | |
| L13 | Other bullous disorders | | |
| L28 | Lichen simplex chronicus and prurigo | | |
| L301 | Dyshidrosis [pompholyx] | | |
| L430 | Hypertrophic lichen planus | | |
| L431 | Bullous lichen planus | | |
| L433 | Subacute (active) lichen planus | | |
| L438 | Other lichen planus | | |
| L439 | Lichen planus, unspecified | | |
| L508 | Other urticaria | | |
| L581 | Chronic radiodermatitis | | |
| L85 | Other epidermal thickening | | |
| Q80 | Congenital ichthyosis | | |
| Q81 | Epidermolysis bullosa | | |
| Q821 | Xeroderma pigmentosum | | |
| Q822 | Mastocytosis | | |
| Q829 | Congenital malformation of skin, unspecified | | |
| **PARKINSON AND PARKINSONISM** | | | |
| **Included ICD-10 codes and labels** | | | |
| G20 | Parkinson disease | | |
| G211 | | Other drug-induced secondary parkinsonism | |
| G212 | | Secondary parkinsonism due to other external agents | |
| G213 | | Postencephalitic parkinsonism | |
| G214 | | Vascular parkinsonism | |
| G218 | | Other secondary parkinsonism | |
| G219 | | Secondary parkinsonism, unspecified | |
| G22 | Parkinsonism in diseases classified elsewhere | | |
| G23 | Other degenerative diseases of basal ganglia | | |
| **PERIPHERAL NEUROPATHY** | | | |
| **Included ICD-10 codes and labels** | | | |
| B91 | Sequelae of poliomyelitis | | |
| G14 | Postpolio syndrome | | |
| G54 | Nerve root and plexus disorders | | |
| G55 | Nerve root and plexus compressions in diseases classified elsewhere | | |
| G56 | Mononeuropathies of upper limb | | |
| G57 | Mononeuropathies of lower limb | | |
| G58 | Other mononeuropathies | | |
| G59 | Mononeuropathy in diseases classified elsewhere | | |
| G60 | Hereditary and idiopathic neuropathy | | |
| G628 | Other specified polyneuropathies | | |
| G629 | Polyneuropathy, unspecified | | |
| G630 | | Polyneuropathy in infectious and parasitic diseases classified elsewhere | |
| G632 | | Diabetic polyneuropathy | |
| G633 | | Polyneuropathy in other endocrine and metabolic diseases | |
| G634 | | Polyneuropathy in nutritional deficiency | |
| G635 | | Polyneuropathy in systemic connective tissue disorders | |
| G636 | | Polyneuropathy in other musculoskeletal disorders | |
| G638 | | Polyneuropathy in other diseases classified elsewhere | |
| M472 | Other spondylosis with radiculopathy | | |
| M531 | Cervicobrachial syndrome | | |
| M541 | Radiculopathy | | |
| **PERIPHERAL VASCULAR DISEASE** | | | |
| **Included ICD-10 codes and labels** | | | |
| I702 | Atherosclerosis of arteries of extremities | | |
| I730 | Raynaud syndrome | | |
| I739 | Peripheral vascular disease, unspecified | | |
| I792 | Peripheral angiopathy in diseases classified elsewhere | | |
| I798 | Other disorders of arteries, arterioles and capillaries in diseases classified elsewhere | | |
| **PROSTATE DISEASES** | | | |
| **Included ICD-10 codes and labels** | | | |
| N40 | Hyperplasia of prostate | | |
| N411 | Chronic prostatitis | | |
| N418 | Other inflammatory diseases of prostate | | |
| **SCHIZOPHRENIA AND DELUSIONAL DISEASES** | | | |
| **Included ICD-10 codes and labels** | | | |
| F20 | Schizophrenia | | |
| F22 | Persistent delusional disorders | | |
| F24 | Induced delusional disorder | | |
| F25 | Schizoaffective disorders | | |
| F28 | Other nonorganic psychotic disorders | | |
| **SLEEP DISORDERS** | | | |
| **Included ICD-10 codes and labels** | | | |
| F510 | Nonorganic insomnia | | |
| F511 | Nonorganic hypersomnia | | |
| F512 | Nonorganic disorder of the sleep-wake schedule | | |
| F513 | Sleepwalking [somnambulism] | | |
| G47 | Sleep disorders | | |
| **SOLID NEOPLASMS** | | | |
| **Included ICD-10 codes and labels** | | | |
| C00 | Malignant neoplasm of lip | | |
| C01 | Malignant neoplasm of base of tongue | | |
| C02 | Malignant neoplasm of other and unspecified parts of tongue | | |
| C03 | Malignant neoplasm of gum | | |
| C04 | Malignant neoplasm of floor of mouth | | |
| C05 | Malignant neoplasm of palate | | |
| C06 | Malignant neoplasm of other and unspecified parts of mouth | | |
| C07 | Malignant neoplasm of parotid gland | | |
| C08 | Malignant neoplasm of other and unspecified major salivary glands | | |
| C09 | Malignant neoplasm of tonsil | | |
| C10 | Malignant neoplasm of oropharynx | | |
| C11 | Malignant neoplasm of nasopharynx | | |
| C12 | Malignant neoplasm of piriform sinus | | |
| C13 | Malignant neoplasm of hypopharynx | | |
| C14 | Malignant neoplasm of other and ill-defined sites in the lip, oral cavity and pharynx | | |
| C15 | Malignant neoplasm of oesophagus | | |
| C16 | Malignant neoplasm of stomach | | |
| C17 | Malignant neoplasm of small intestine | | |
| C18 | Malignant neoplasm of colon | | |
| C19 | Malignant neoplasm of rectosigmoid junction | | |
| C20 | Malignant neoplasm of rectum | | |
| C21 | Malignant neoplasm of anus and anal canal | | |
| C22 | Malignant neoplasm of liver and intrahepatic bile ducts | | |
| C23 | Malignant neoplasm of gallbladder | | |
| C24 | Malignant neoplasm of other and unspecified parts of biliary tract | | |
| C25 | Malignant neoplasm of pancreas | | |
| C26 | Malignant neoplasm of other and ill-defined digestive organs | | |
| C30 | Malignant neoplasm of nasal cavity and middle ear | | |
| C31 | Malignant neoplasm of accessory sinuses | | |
| C32 | Malignant neoplasm of larynx | | |
| C33 | Malignant neoplasm of trachea | | |
| C34 | Malignant neoplasm of bronchus and lung | | |
| C37 | Malignant neoplasm of thymus | | |
| C38 | Malignant neoplasm of heart, mediastinum and pleura | | |
| C39 | Malignant neoplasm of other and ill-defined sites in the respiratory system and intrathoracic organs | | |
| C40 | Malignant neoplasm of bone and articular cartilage of limbs | | |
| C41 | Malignant neoplasm of bone and articular cartilage of other and unspecified sites | | |
| C43 | Malignant melanoma of skin | | |
| C44 | Other malignant neoplasms of skin | | |
| C45 | Mesothelioma | | |
| C46 | Kaposi sarcoma | | |
| C47 | Malignant neoplasm of peripheral nerves and autonomic nervous system | | |
| C48 | Malignant neoplasm of retroperitoneum and peritoneum | | |
| C49 | Malignant neoplasm of other connective and soft tissue | | |
| C50 | Malignant neoplasm of breast | | |
| C51 | Malignant neoplasm of vulva | | |
| C52 | Malignant neoplasm of vagina | | |
| C53 | Malignant neoplasm of cervix uteri | | |
| C54 | Malignant neoplasm of corpus uteri | | |
| C55 | Malignant neoplasm of uterus, part unspecified | | |
| C56 | Malignant neoplasm of ovary | | |
| C57 | Malignant neoplasm of other and unspecified female genital organs | | |
| C58 | Malignant neoplasm of placenta | | |
| C60 | Malignant neoplasm of penis | | |
| C61 | Malignant neoplasm of prostate | | |
| C62 | Malignant neoplasm of testis | | |
| C63 | Malignant neoplasm of other and unspecified male genital organs | | |
| C64 | Malignant neoplasm of kidney, except renal pelvis | | |
| C65 | Malignant neoplasm of renal pelvis | | |
| C66 | Malignant neoplasm of ureter | | |
| C67 | Malignant neoplasm of bladder | | |
| C68 | Malignant neoplasm of other and unspecified urinary organs | | |
| C69 | Malignant neoplasm of eye and adnexa | | |
| C70 | Malignant neoplasm of meninges | | |
| C71 | Malignant neoplasm of brain | | |
| C72 | Malignant neoplasm of spinal cord, cranial nerves and other parts of central nervous system | | |
| C73 | Malignant neoplasm of thyroid gland | | |
| C74 | Malignant neoplasm of adrenal gland | | |
| C75 | Malignant neoplasm of other endocrine and related structures | | |
| C76 | Malignant neoplasm of other and ill-defined sites | | |
| C77 | Secondary and unspecified malignant neoplasm of lymph nodes | | |
| C78 | Secondary malignant neoplasm of respiratory and digestive organs | | |
| C79 | Secondary malignant neoplasm of other and unspecified sites | | |
| C80 | Malignant neoplasm without specification of site | | |
| C97 | Malignant neoplasms of independent (primary) multiple sites | | |
| D00 | Carcinoma in situ of oral cavity, oesophagus and stomach | | |
| D01 | Carcinoma in situ of other and unspecified digestive organs | | |
| D02 | Carcinoma in situ of middle ear and respiratory system | | |
| D03 | Melanoma in situ | | |
| D04 | Carcinoma in situ of skin | | |
| D05 | Carcinoma in situ of breast | | |
| D06 | Carcinoma in situ of cervix uteri | | |
| D07 | Carcinoma in situ of other and unspecified genital organs | | |
| D09 | Carcinoma in situ of other and unspecified sites | | |
| D320 | Benign neoplasm: Cerebral meninges | | |
| D321 | Benign neoplasm: Spinal meninges | | |
| D329 | Benign neoplasm: Meninges, unspecified | | |
| D330 | Benign neoplasm: Brain, supratentorial | | |
| D331 | Benign neoplasm: Brain, infratentorial | | |
| D332 | Benign neoplasm: Brain, unspecified | | |
| D333 | Benign neoplasm: Cranial nerves | | |
| D334 | Benign neoplasm: Spinal cord | | |
| Q85 | Phakomatoses, not elsewhere classified | | |
| **THYROID DISEASES** | | | |
| **Included ICD-10 codes and labels** | | | |
| E00 | Congenital iodine-deficiency syndrome | | |
| E01 | Iodine-deficiency-related thyroid disorders and allied conditions | | |
| E02 | Subclinical iodine-deficiency hypothyroidism | | |
| E030 | Congenital hypothyroidism with diffuse goitre | | |
| E031 | Congenital hypothyroidism without goitre | | |
| E032 | Hypothyroidism due to medicaments and other exogenous substances | | |
| E033 | Postinfectious hypothyroidism | | |
| E034 | Atrophy of thyroid (acquired) | | |
| E038 | Other specified hypothyroidism | | |
| E039 | Hypothyroidism, unspecified | | |
| E05 | Thyrotoxicosis [hyperthyroidism] | | |
| E062 | Chronic thyroiditis with transient thyrotoxicosis | | |
| E063 | Autoimmune thyroiditis | | |
| E065 | Other chronic thyroiditis | | |
| E07 | Other disorders of thyroid | | |
| E350 | Disorders of thyroid gland in diseases classified elsewhere | | |
| E890 | Postprocedural hypothyroidism | | |
| **VENOUS AND LYMPHATIC DISEASES** | | | |
| **Included ICD-10 codes and labels** | | | |
| I780 | Hereditary haemorrhagic telangiectasia | | |
| I83 | Varicose veins of lower extremities | | |
| I87 | Other disorders of veins | | |
| I89 | Other noninfective disorders of lymphatic vessels and lymph nodes | | |
| I972 | Postmastectomy lymphoedema syndrome | | |
| Q820 | Hereditary lymphoedema | | |

**Table S2.** Drugs used to define chronic disease groups

| **Disease Group** | **ATC Codes** | **Description of ATC Codes** |
| --- | --- | --- |
| Anemia | B03XA, B03A | Antianemic preparations (including erythropoiesis-stimulating agents and iron supplements). |
| Asthma | R03DC, R03BC | Inhaled corticosteroids (R03DC), inhaled beta-2 agonists (R03BC). |
| Autoimmune diseases | D05 | Antipsoriatics for systemic use. |
| Chronic infectious diseases | J04A (excluding J04AB01, J04AB02, J04AB03, J04AC) | Drugs for treatment of tuberculosis (excluding combination products or other specific antitubercular drugs). |
| Chronic pancreas, biliary and gallbladder disease | A09AA02 | Digestive enzyme preparations for pancreatic insufficiency (e.g., pancreatin). |
| COPD | R03BB | Inhaled anticholinergic agents used for respiratory conditions. |
| Dementia | N06DA, N06DX01 | Cholinesterase inhibitors and other drugs for dementia. |
| Diabetes | A10 | Drugs used in diabetes, including insulin and oral hypoglycemic agents. |
| Esophagus stomach diseases | A02BX | Proton pump inhibitors and other acid-related disorders drugs. |
| Glaucoma | S01ED | Antiglaucoma preparations and miotics. |
| Inflammatory arthropathies | M01CB | Specific anti-inflammatory and anti-rheumatic drugs for musculoskeletal disorders. |
| Inflammatory bowel diseases | A07E | Drugs for inflammatory bowel disease, such as aminosalicylates. |
| Ischemic heart disease | C01DA, C01EB18 | Antianginal drugs and specific coronary vasodilators. |
| Migraine, facial pain | N02C | Drugs for migraine, including triptans and ergot derivatives. |
| Osteoporosis | M05BA, M05BB, M05BX03, M05BX53 | Drugs for treatment of bone diseases, including bisphosphonates and monoclonal antibodies for osteoporosis. |
| Other psychiatric and behavioral diseases | N07BB | Drugs used in the treatment of substance dependence (e.g., nicotine dependence). |
| Parkinson and parkinsonism | N04BA, N04BX, N04BC | Antiparkinson drugs, including dopaminergic agents and anticholinergics. |
| Peripheral vascular diseases | B01AC23 | Antithrombotic agents, specifically used for peripheral arterial disease. |
| Prostate diseases | G04C (excluding G04CB) | Drugs used for benign prostatic hyperplasia (excluding those specifically for bladder and urethral function). |
| Thyroid diseases | H03AA, H03B | Drugs for thyroid therapy, including thyroxine and antithyroid agents. |

**Table S3.** Prevalence of chronic conditions in total sample and age strata

|  |  | **Age stratum** | | |
| --- | --- | --- | --- | --- |
| **Chronic condition** | **Total** | **18-64** | **65-74** | **≥75** |
| Allergy | 6,1 | 7,3 | 3,8 | 2,8 |
| Anemia | 9,3 | 9,0 | 6,9 | 14,2 |
| Asthma | 7,4 | 7,5 | 7,2 | 7,1 |
| Atrial fibrillation | 4,5 | 1,3 | 7,7 | 17,7 |
| Autoimmune diseases | 5,8 | 4,8 | 7,3 | 9,1 |
| Blindness, visual impairment | 0,7 | 0,4 | 0,9 | 2,2 |
| Blood and blood forming organ diseases | 1,6 | 1,6 | 1,6 | 1,9 |
| Bradycardias and conduction diseases | 1,0 | 0,3 | 1,4 | 4,2 |
| Cardiac valve diseases | 1,5 | 0,6 | 2,3 | 5,4 |
| Cataract and other lens diseases | 8,4 | 2,0 | 16,8 | 31,9 |
| Cerebrovascular disease | 3,8 | 1,7 | 6,4 | 12,2 |
| Chromosomal abnormalities | 0,1 | 0,1 | 0,0 | 0,0 |
| Chronic infectious diseases | 4,2 | 3,7 | 6,2 | 4,6 |
| Chronic kidney diseases | 1,7 | 1,2 | 1,6 | 4,1 |
| Chronic liver diseases | 1,2 | 1,3 | 1,2 | 0,6 |
| Chronic pancreas, biliary tract and gallbladder diseases | 2,9 | 2,8 | 3,0 | 3,1 |
| Chronic ulcer of the skin | 2,1 | 1,3 | 2,6 | 5,5 |
| Colitis and related diseases | 11,3 | 10,1 | 12,1 | 16,8 |
| COPD, emphysema, chronic bronchitis | 4,2 | 2,1 | 7,9 | 10,3 |
| Deafness, hearing impairment | 5,8 | 3,0 | 9,4 | 16,2 |
| Dementia | 1,1 | 0,1 | 1,2 | 6,5 |
| Depression and mood diseases | 13,1 | 15,2 | 7,8 | 9,2 |
| Diabetes | 14,2 | 12,3 | 19,3 | 17,8 |
| Dorsopathies | 7,4 | 6,6 | 7,7 | 10,9 |
| Dyslipidemia | 10,8 | 7,3 | 20,2 | 17,1 |
| Ear, nose, throat diseases | 6,5 | 6,8 | 6,0 | 5,7 |
| Epilepsy | 0,9 | 0,8 | 0,9 | 1,1 |
| Esophagus, stomach and duodenum diseases | 7,1 | 6,8 | 7,7 | 8,1 |
| Glaucoma | 2,7 | 0,8 | 4,4 | 10,6 |
| Heart failure | 3,2 | 0,9 | 4,3 | 13,9 |
| Hematological neoplasms | 0,9 | 0,6 | 1,4 | 1,9 |
| Hypertension | 34,1 | 23,2 | 56,4 | 63,1 |
| Inflammatory arthropathies | 5,9 | 5,2 | 6,8 | 8,5 |
| Inflammatory bowel diseases | 2,2 | 2,4 | 1,9 | 1,7 |
| Ischemic heart disease | 8,2 | 3,7 | 14,0 | 24,6 |
| Migraine and facial pain syndromes | 7,3 | 8,8 | 4,2 | 3,2 |
| Multiple sclerosis | 0,3 | 0,4 | 0,3 | 0,1 |
| Neurotic, stress-related and somatoform diseases | 16,8 | 20,7 | 8,2 | 7,5 |
| Obesity | 6,0 | 6,9 | 5,0 | 2,3 |
| Osteoarthritis and other degenerative joint diseases | 11,0 | 6,2 | 19,9 | 24,6 |
| Osteoporosis | 4,0 | 1,6 | 6,9 | 13,0 |
| Other cardiovascular diseases | 2,7 | 1,3 | 4,1 | 8,3 |
| Other digestive diseases | 1,2 | 1,1 | 1,1 | 1,5 |
| Other eye diseases | 8,1 | 4,7 | 12,5 | 21,2 |
| Other genitourinary diseases | 9,8 | 8,0 | 13,8 | 13,6 |
| Other metabolic diseases | 3,2 | 3,1 | 3,0 | 4,1 |
| Other musculoskeletal and joint diseases | 15,9 | 15,3 | 17,9 | 16,5 |
| Other neurological diseases | 2,4 | 1,9 | 3,2 | 4,2 |
| Other psychiatric and behavioral diseases | 6,5 | 7,0 | 4,7 | 6,1 |
| Other respiratory diseases | 0,6 | 0,4 | 0,9 | 1,4 |
| Other skin diseases | 1,4 | 1,2 | 1,7 | 1,9 |
| Parkinson and parkinsonism | 1,4 | 0,8 | 2,3 | 3,7 |
| Peripheral neuropathy | 5,0 | 4,7 | 5,2 | 6,5 |
| Peripheral vascular disease | 1,3 | 0,6 | 2,0 | 3,9 |
| Prostate diseases | 5,3 | 2,8 | 10,3 | 12,0 |
| Schizophrenia and delusional diseases | 0,6 | 0,7 | 0,5 | 0,4 |
| Sleep disorders | 6,2 | 6,1 | 6,4 | 6,4 |
| Solid neoplasms | 9,2 | 5,1 | 16,1 | 22,0 |
| Thyroid diseases | 9,9 | 8,3 | 11,8 | 15,7 |
| Venous and lymphatic diseases | 2,9 | 1,9 | 4,0 | 6,5 |

**Table S4.** Conditions characterizing each multimorbidity pattern among 75+ year olds (shaded meet both overexpression criteria).

| **Multimorbidity**  **Pattern** | **Disease** | **Exclusivity (%)** | **Observed/expected**  **Ratio** | **Prevalence (%)** |
| --- | --- | --- | --- | --- |
| **Unspecific (complex)** | Hematological neoplasms | 55 | 2,3 | 4,7 |
| **(n=18,785)** | Osteoporosis | 39 | 1,7 | 22,7 |
|  | Autoimmune diseases | 36 | 1,5 | 14,6 |
|  | Chronic infectious diseases | 33 | 1,4 | 6,8 |
|  | Inflammatory arthropathies | 30 | 1,3 | 11,5 |
|  | Osteoarthritis and other degenerative joint diseases | 30 | 1,3 | 33,1 |
|  | Other musculoskeletal and joint diseases | 30 | 1,3 | 22,6 |
|  | Peripheral neuropathy | 31 | 1,3 | 9 |
|  | Solid neoplasms | 31 | 1,3 | 30,4 |
|  | Dorsopathies | 29 | 1,2 | 14,1 |
|  | Venous and lymphatic diseases | 30 | 1,2 | 8,7 |
|  | Ear, nose, throat diseases | 25 | 1,1 | 6,5 |
|  | Esophagus, stomach and duodenum diseases | 26 | 1,1 | 9,4 |
|  | Migraine and facial pain syndromes | 26 | 1,1 | 3,8 |
|  | Other genitourinary diseases | 27 | 1,1 | 16,2 |
|  | Other skin diseases | 25 | 1,1 | 2,2 |
|  | Prostate diseases | 25 | 1,1 | 13,3 |
|  | Thyroid diseases | 27 | 1,1 | 18,7 |
|  | Allergy | 25 | 1 | 3,1 |
| **Cardiovascular** | Bradycardias and conduction diseases | 73 | 5,5 | 24,4 |
| **(n=10,559)** | Heart failure | 67 | 5,1 | 75,3 |
|  | Atrial fibrillation | 49 | 3,7 | 69 |
|  | Cardiac valve diseases | 49 | 3,7 | 21,4 |
|  | Chronic kidney diseases | 44 | 3,3 | 14,6 |
|  | Other cardiovascular diseases | 36 | 2,7 | 24,2 |
|  | Ischemic heart disease | 30 | 2,3 | 59,6 |
|  | Anemia | 29 | 2,2 | 32,7 |
|  | Inflammatory arthropathies | 27 | 2,1 | 18,6 |
|  | COPD, emphysema, chronic bronchitis | 26 | 2 | 21,8 |
| **Eye** | Cataract and other lens diseases | 53 | 2,6 | 89,3 |
| **(n=16,047)** | Other eye diseases | 51 | 2,5 | 56,8 |
|  | Blindness, visual impairment | 42 | 2,1 | 4,9 |
|  | Glaucoma | 43 | 2,1 | 23,9 |
|  | Deafness, hearing impairment | 26 | 1,3 | 21,9 |
| **Vascular** | Peripheral vascular disease | 60 | 23,9 | 100 |
| **(n=2,000)** | Other cardiovascular diseases | 28 | 11 | 97 |
|  | Chronic ulcer of the skin | 11 | 4,5 | 26,6 |
|  | Venous and lymphatic diseases | 6 | 2,2 | 15,3 |
|  | Chronic kidney diseases | 5 | 2,1 | 9,2 |
| **Multisystem** | Neurotic, stress-related and somatoform diseases | 44 | 4 | 32,3 |
| **(n=8,647)** | Sleep disorders | 41 | 3,8 | 25,8 |
|  | Depression and mood diseases | 40 | 3,6 | 35,5 |
|  | Migraine and facial pain syndromes | 33 | 3,1 | 10,6 |
|  | Other neurological diseases | 34 | 3,1 | 13,8 |
|  | Dorsopathies | 32 | 3 | 34,4 |
|  | Parkinson and parkinsonism | 32 | 3 | 11,9 |
|  | Allergy | 31 | 2,9 | 8,7 |
|  | Peripheral neuropathy | 31 | 2,9 | 19,8 |
|  | Ear, nose, throat diseases | 31 | 2,8 | 17 |
|  | Other metabolic diseases | 30 | 2,8 | 12,1 |
|  | Esophagus, stomach and duodenum diseases | 29 | 2,7 | 22,9 |
|  | Asthma | 27 | 2,5 | 19,1 |
|  | Colitis and related diseases | 27 | 2,5 | 44,7 |
|  | Other genitourinary diseases | 26 | 2,4 | 34,1 |
|  | Other musculoskeletal and joint diseases | 26 | 2,3 | 41,1 |
|  | Other skin diseases | 25 | 2,3 | 4,7 |
|  | Blindness, visual impairment | 24 | 2,2 | 5,3 |
|  | Obesity | 24 | 2,2 | 5,5 |
|  | Chronic pancreas, biliary tract and gallbladder diseases | 22 | 2,1 | 6,9 |
|  | Osteoporosis | 23 | 2,1 | 28,7 |
| **Unspecific (Risk factor)** | Diabetes | 43 | 1,8 | 34,5 |
| **(n=18,626)** | Dyslipidemia | 43 | 1,8 | 33,1 |
|  | Obesity | 34 | 1,4 | 3,6 |
|  | Hypertension | 30 | 1,3 | 83,2 |
|  | Ischemic heart disease | 30 | 1,3 | 33,6 |
|  | Cerebrovascular disease | 29 | 1,2 | 16,1 |
| **Dementia** | Dementia | 65 | 10,8 | 74,5 |
| **(n=4,823)** | Other psychiatric and behavioral diseases | 57 | 9,3 | 60,8 |
|  | Depression and mood diseases | 14 | 2,2 | 21,9 |

**Table S5.** Description of patterns (75+ year olds)

|  | **No MM** | **Cardiovascular** | **Eye** | **Vascular** | **Unspecific (Complex)** | **Multisystem** | **Unspecific (RF)** | **Dementia** |
| --- | --- | --- | --- | --- | --- | --- | --- | --- |
|  | **N=5,502** | **N=10,559** | **N=16,047** | **N=2,000** | **N=18,785** | **N=8,647** | **N=18,626** | **N=4,823** |
| **Age** | 80.5 [±4.6] | 83.6 [±5.4] | 81.3 [±4.8] | 82.4 [±5.1] | 80.8 [±4.6] | 81.9 [±5.0] | 80.8 [±4.6] | 82.8 [±5.2] |
| **Sex (female)** | 59.9 (3,297) | 52.3 (5,525) | 65.1 (10,442) | 59.2 (1,185) | 64.9 (12,196) | 78.9 (6,819) | 54.6 (10,164) | 58.3 (2,813) |
| **Number of conditions** | 0.8 [±0.4] | 9.0 [±2.8] | 5.7 [±2.0] | 9.0 [±3.1] | 4.7 [±1.9] | 10.4 [±2.6] | 4.1 [±1.7] | 5.8 [±2.4] |
| **eGFR** | 61 [±12] | 50 [±15] | 60 [±12] | 53 [±16] | 61 [±13] | 59 [±13] | 58 [±13] | 59 [±13] |
| **eGFR <60** | 43.8 (2,409) | 72.8 (7,686) | 47.4 (7,613) | 64.3 (1,287) | 44.4 (8,348) | 49.1 (4,245) | 52.0 (9,690) | 50.7 (2,447) |
| **Outcome (Incident A2 or A3)** |  |  |  |  |  |  |  |  |
| Administrative censoring | 44.2 (2,434) | 25.8 (2,726) | 62.1 (9,971) | 24.1 (482) | 46.4 (8,719) | 57.6 (4,977) | 41.3 (7,684) | 27.1 (1,307) |
| Incident A2 | 8.5 (465) | 10.3 (1,088) | 8.0 (1,283) | 14.1 (281) | 9.7 (1,820) | 6.7 (581) | 13.8 (2,564) | 4.6 (224) |
| Death | 44.1 (2,425) | 62.3 (6,581) | 28.0 (4,486) | 59.6 (1,192) | 41.5 (7,804) | 34.2 (2,956) | 42.4 (7,895) | 66.0 (3,181) |
| RRT | 0.0 (2) | 0.1 (6) | 0.0 (5) | 0.1 (2) | 0.0 (3) | 0.0 (1) | 0.0 (3) | 0.0 (0) |
| Emigration | 3.2 (176) | 1.5 (158) | 1.9 (302) | 2.1 (43) | 2.3 (439) | 1.5 (132) | 2.6 (480) | 2.3 (111) |
| **Outcome (Incident A3)** |  |  |  |  |  |  |  |  |
| Administrative censoring | 46.8 (2,575) | 27.8 (2,939) | 65.3 (10,480) | 26.4 (528) | 49.3 (9,256) | 60.0 (5,192) | 45.5 (8,476) | 28.0 (1,351) |
| Incident A3 | 2.1 (118) | 2.4 (251) | 1.8 (293) | 3.4 (68) | 2.6 (485) | 1.5 (127) | 3.1 (583) | 1.2 (57) |
| Death | 47.6 (2,620) | 68.1 (7,193) | 30.8 (4,948) | 67.8 (1,357) | 45.7 (8,576) | 36.9 (3,188) | 48.6 (9,053) | 68.5 (3,303) |
| RRT | 0.1 (4) | 0.1 (9) | 0.0 (5) | 0.1 (2) | 0.0 (6) | 0.0 (3) | 0.0 (5) | 0.0 (0) |
| Emigration | 3.4 (185) | 1.6 (167) | 2.0 (321) | 2.2 (45) | 2.5 (462) | 1.6 (137) | 2.7 (509) | 2.3 (112) |
| Data are presented as mean [±SD] for continuous measures, and % (n) for categorical measures. | | | | | | | | |

**Table S6.** Albuminuria testing rates during follow-up stratified by age group and multimorbidity pattern

| Age group | Multimorbidity pattern | Percent ever tested during follow-up | Annual testing rate |
| --- | --- | --- | --- |
| **75 and above** | *Total stratum* | 67 | 0.5 |
|  | Vascular | 65 | 0.6 |
|  | Unspecific (risk factors) | 75 | 0.6 |
|  | Cardiovascular | 61 | 0.6 |
|  | Multisystem | 63 | 0.6 |
|  | Eye | 65 | 0.5 |
|  | Unspecific (complex) | 69 | 0.6 |
|  | Dementia | 45 | 0.3 |
|  | No multimorbidity | 68 | 0.4 |
| **65 to 74** | *Total stratum* | 74 | 0.6 |
|  | Cardiovascular | 75 | 0.8 |
|  | Unspecific (risk factors) | 80 | 0.7 |
|  | Multisystem | 73 | 0.7 |
|  | Eye | 70 | 0.6 |
|  | Mental health | 66 | 0.6 |
|  | Unspecific (Complex) | 70 | 0.6 |
|  | No multimorbidity | 69 | 0.4 |
| **18 to 64** | *Total stratum* | 59 | 0.6 |
|  | Cardiometabolic | 76 | 0.7 |
|  | Neuro-musculoskeletal | 70 | 0.6 |
|  | Unspecific | 61 | 0.5 |
|  | Mental health | 54 | 0.3 |
|  | No multimorbidity | 50 | 0.3 |

**Table S7.** Sensitivity analyses for primary outcome (time to A2+)

| Age group | Multimorbidity pattern | Adjusted for age | Adjusted for baseline UACR | Adjusted for uncertainty in class assignment |
| --- | --- | --- | --- | --- |
| **75 and above** | *Total stratum* |  |  |  |
|  | Unspecific (complex) | **1.42 [1.28,1.57]** | **1.43 [1.29,1.58]** | **1.44 [1.29,1.61]** |
|  | Cardiometabolic | **1.79 [1.60,2.00]** | **1.75 [1.57,1.95]** | **1.82 [1.62,2.05]** |
|  | Eye | **1.43 [1.29,1.59]** | **1.47 [1.33,1.64]** | **1.46 [1.28,1.66]** |
|  | Vascular | **2.39 [2.06,2.78]** | **2.27 [1.96,2.64]** | **2.39 [2.04,2.80]** |
|  | Multisystem | **1.44 [1.27,1.62]** | **1.45 [1.28,1.63]** | **1.47 [1.27,1.71]** |
|  | Unspecific (risk factors) | **1.85 [1.68,2.04]** | **1.80 [1.63,1.99]** | **1.89 [1.50,2.38]** |
|  | Dementia | 0.93 [0.79,1.09] | 0.93 [0.80,1.10] | 0.94 [0.70,1.25] |
|  | No multimorbidity | **Ref.** | Ref. | Ref. |
| **65 to 74** | *Total stratum* |  |  |  |
|  | Unspecific (risk factors) | **2.06 [1.93,2.21]** | **1.96 [1.83,2.10]** | **2.07 [1.70,2.53]** |
|  | Cardiovascular | **3.14 [2.90,3.40]** | **2.87 [2.65,3.11]** | **3.03 [2.68,3.42]** |
|  | Eye | **1.75 [1.60,1.92]** | **1.72 [1.57,1.88]** | **1.71 [1.48,1.97]** |
|  | Unspecific (Complex) | **1.37 [1.28,1.48]** | **1.34 [1.24,1.44]** | **1.38 [1.14,1.66]** |
|  | Mental health | **1.62 [1.44,1.82]** | **1.58 [1.41,1.78]** | **1.63 [1.37,1.93]** |
|  | Musculoskeletal & respiratory | **2.03 [1.83,2.25]** | **1.92 [1.73,2.12]** | **2.00 [1.74,2.29]** |
|  | No multimorbidity | Ref. | Ref. | Ref. |
| **18 to 64** | *Total stratum* |  |  |  |
|  | Unspecific | **1.93 [1.85,2.01]** | **1.94 [1.86,2.02]** | **2.00 [1.80,2.21]** |
|  | Cardiovascular | **3.14 [3.02,3.27]** | **3.13 [3.00,3.26]** | **3.37 [2.48,4.58]** |
|  | Mental health | **1.60 [1.52,1.68]** | **1.61 [1.53,1.70]** | **1.66 [1.54,1.79]** |
|  | Musculoskeletal & respiratory | **2.93 [2.74,3.14]** | **2.94 [2.75,3.15]** | **3.17 [2.53,3.97]** |
|  | No multimorbidity | Ref. | Ref. | Ref. |
|  |  |  |  |  |
|  |  |  |  |  |
|  |  |  |  |  |

| Age group | Multimorbidity pattern | Adjusted for age | Adjusted for baseline UACR | Adjusted for uncertainty in class assignment |
| --- | --- | --- | --- | --- |
| **75 and above** | *Total stratum* |  |  |  |
|  | Unspecific (complex) | **1.55 [1.27,1.90]** | **1.50 [1.23,1.83]** | **1.47 [1.18,1.82]** |
|  | Cardiometabolic | **1.62 [1.29,2.02]** | **1.41 [1.13,1.77]** | **1.50 [1.17,1.92]** |
|  | Eye | **1.40 [1.13,1.74]** | **1.32 [1.07,1.64]** | **1.31 [1.04,1.66]** |
|  | Vascular | **2.25 [1.67,3.05]** | **1.94 [1.44,2.63]** | **2.03 [1.46,2.84]** |
|  | Multisystem | **1.43 [1.11,1.84]** | **1.29 [1.00,1.66]** | 1.31 [0.91,1.90] |
|  | Unspecific (risk factors) | **1.62 [1.33,1.97]** | **1.51 [1.24,1.84]** | **1.62 [1.24,2.12]** |
|  | Dementia | 1.05 [0.77,1.44] | 0.94 [0.68,1.28] | 0.92 [0.61,1.38] |
|  | No multimorbidity | Ref. | Re. | Ref. |
| **65 to 74** | *Total stratum* |  |  |  |
|  | Unspecific (risk factors) | **1.80 [1.56,2.07]** | **1.62 [1.40,1.86]** | **1.76 [1.48,2.09]** |
|  | Cardiovascular | **3.13 [2.65,3.68]** | **2.63 [2.23,3.09]** | **2.73 [2.29,3.26]** |
|  | Eye | **1.86 [1.54,2.25]** | **1.62 [1.34,1.96]** | **1.65 [1.31,2.07]** |
|  | Unspecific (Complex) | **1.53 [1.32,1.78]** | **1.43 [1.23,1.66]** | **1.49 [1.26,1.77]** |
|  | Mental health | **1.73 [1.35,2.22]** | **1.60 [1.24,2.05]** | **1.69 [1.28,2.21]** |
|  | Musculoskeletal & respiratory | **2.47 [2.01,3.03]** | **2.11 [1.72,2.59]** | **2.10 [1.60,2.76]** |
|  | No multimorbidity | Ref. | Ref. | Ref. |
| **18 to 64** | *Total stratum* |  |  |  |
|  | Unspecific | **2.25 [2.06,2.46]** | **2.15 [1.97,2.35]** | **2.21 [1.95,2.51]** |
|  | Cardiovascular | **3.22 [2.95,3.52]** | **2.87 [2.63,3.14]** | **3.15 [2.44,4.05]** |
|  | Mental health | **1.62 [1.45,1.82]** | **1.55 [1.38,1.74]** | **1.65 [1.43,1.89]** |
|  | Musculoskeletal & respiratory | **3.29 [2.84,3.81]** | **2.89 [2.49,3.35]** | **3.12 [2.34,4.16]** |
|  | No multimorbidity | Ref. | Ref. | Ref. |
|  |  |  |  |  |
|  |  |  |  |  |
|  |  |  |  |  |

**Table S8.** Sensitivity analyses for secondary outcome (time to A3)

**Table S9**. Conditions characterizing each multimorbidity pattern among 65 to 74 year olds (shaded meet both overexpression criteria).

| **Multimorbidity**  **Pattern** | **Disease** | **Exclusivity (%)** | **Observed/expected**  **Ratio** | **Prevalence (%)** |
| --- | --- | --- | --- | --- |
| **Unspecific (risk factors)** | Dyslipidemia | 61 | 1,7 | 39,3 |
| **(n=37,297)** | Diabetes | 59 | 1,6 | 35 |
|  | Obesity | 53 | 1,5 | 8,6 |
|  | Hypertension | 50 | 1,4 | 85,3 |
|  | Ischemic heart disease | 45 | 1,3 | 19,9 |
|  | Cerebrovascular disease | 42 | 1,2 | 8,7 |
|  | Prostate diseases | 31 | 0,9 | 9,9 |
|  | Chronic infectious diseases | 27 | 0,8 | 5,3 |
|  | Thyroid diseases | 28 | 0,8 | 10,5 |
|  | Atrial fibrillation | 26 | 0,7 | 6,4 |
| **Cardiovascular** | Heart failure | 80 | 9,3 | 46,5 |
| **(n=8,973)** | Peripheral vascular disease | 78 | 9,1 | 21,2 |
|  | Other cardiovascular diseases | 69 | 8,1 | 37,9 |
|  | Cardiac valve diseases | 56 | 6,5 | 16,8 |
|  | Atrial fibrillation | 44 | 5,1 | 44,9 |
|  | Ischemic heart disease | 28 | 3,3 | 51,6 |
|  | Chronic ulcer of the skin | 27 | 3,1 | 9,2 |
|  | Anemia | 24 | 2,8 | 22,5 |
|  | Cerebrovascular disease | 22 | 2,6 | 18,9 |
|  | COPD, emphysema, chronic bronchitis | 21 | 2,4 | 21,7 |
| **Eye** | Cataract and other lens diseases | 51 | 4,7 | 89,6 |
| **(n=11,321)** | Glaucoma | 44 | 4 | 20,2 |
|  | Other eye diseases | 42 | 3,9 | 55,4 |
| **Unspecific (Complex)** | Inflammatory bowel diseases | 61 | 1,9 | 4,3 |
| **(n=32,698)** | Osteoporosis | 57 | 1,8 | 14,4 |
|  | Autoimmune diseases | 49 | 1,5 | 12,7 |
|  | Chronic infectious diseases | 44 | 1,4 | 9,7 |
|  | Inflammatory arthropathies | 45 | 1,4 | 11 |
|  | Solid neoplasms | 44 | 1,4 | 25,6 |
|  | Colitis and related diseases | 39 | 1,3 | 17,1 |
|  | Migraine and facial pain syndromes | 39 | 1,3 | 6 |
|  | Osteoarthritis and other degenerative joint diseases | 39 | 1,3 | 28,4 |
|  | Other genitourinary diseases | 40 | 1,3 | 19,7 |
|  | Other musculoskeletal and joint diseases | 40 | 1,3 | 25,6 |
|  | Parkinson and parkinsonism | 40 | 1,3 | 3,3 |
|  | Prostate diseases | 40 | 1,3 | 14,6 |
|  | Allergy | 37 | 1,2 | 5,2 |
|  | Chronic pancreas, biliary tract and gallbladder diseases | 37 | 1,2 | 4,1 |
|  | Dorsopathies | 38 | 1,2 | 10,6 |
|  | Ear, nose, throat diseases | 37 | 1,2 | 8,1 |
|  | Other metabolic diseases | 37 | 1,2 | 4,1 |
|  | Peripheral neuropathy | 37 | 1,2 | 7 |
|  | Venous and lymphatic diseases | 38 | 1,2 | 5,5 |
|  | Anemia | 35 | 1,1 | 9 |
|  | Deafness, hearing impairment | 35 | 1,1 | 11,9 |
|  | Esophagus, stomach and duodenum diseases | 35 | 1,1 | 9,9 |
|  | Thyroid diseases | 34 | 1,1 | 14,3 |
|  | Chronic ulcer of the skin | 32 | 1 | 3 |
|  | Other neurological diseases | 32 | 1 | 3,7 |
|  | Asthma | 29 | 0,9 | 7,7 |
|  | COPD, emphysema, chronic bronchitis | 27 | 0,9 | 7,8 |
| **Mental health** | Depression and mood diseases | 51 | 8,7 | 77,8 |
| **(n=6,089)** | Neurotic, stress-related and somatoform diseases | 46 | 7,9 | 74 |
|  | Other psychiatric and behavioral diseases | 27 | 4,7 | 24,7 |
|  | Sleep disorders | 21 | 3,6 | 26,4 |
|  | Parkinson and parkinsonism | 11 | 2 | 5,1 |
| **Multisystem** | Peripheral neuropathy | 32 | 4,2 | 24,6 |
| **(n=8,016)** | Dorsopathies | 30 | 3,9 | 34,3 |
|  | Allergy | 27 | 3,5 | 15,2 |
|  | Asthma | 26 | 3,4 | 27,9 |
|  | Parkinson and parkinsonism | 25 | 3,2 | 8,5 |
|  | Ear, nose, throat diseases | 24 | 3,1 | 21,2 |
|  | Sleep disorders | 24 | 3,1 | 22,6 |
|  | Migraine and facial pain syndromes | 23 | 3 | 14,4 |
|  | Obesity | 23 | 3 | 17,5 |
|  | Other musculoskeletal and joint diseases | 23 | 3 | 60,3 |
|  | Other neurological diseases | 23 | 3 | 10,8 |
|  | Esophagus, stomach and duodenum diseases | 23 | 2,9 | 25,8 |
|  | Osteoarthritis and other degenerative joint diseases | 21 | 2,8 | 62,4 |
|  | Colitis and related diseases | 19 | 2,5 | 34,6 |
|  | Other eye diseases | 19 | 2,5 | 36 |
|  | Venous and lymphatic diseases | 19 | 2,5 | 11,4 |
|  | Other genitourinary diseases | 19 | 2,4 | 37,7 |
|  | Cataract and other lens diseases | 18 | 2,3 | 44,8 |
|  | Neurotic, stress-related and somatoform diseases | 18 | 2,3 | 21,7 |
|  | Osteoporosis | 18 | 2,3 | 18,2 |
|  | Chronic pancreas, biliary tract and gallbladder diseases | 17 | 2,2 | 7,4 |
|  | COPD, emphysema, chronic bronchitis | 17 | 2,2 | 19,3 |
|  | Depression and mood diseases | 17 | 2,2 | 19,4 |
|  | Other metabolic diseases | 17 | 2,2 | 7,6 |
|  | Chronic ulcer of the skin | 16 | 2,1 | 6,2 |
|  | Anemia | 16 | 2 | 16,1 |
|  | Deafness, hearing impairment | 15 | 2 | 20,8 |

**Table S10.** Description of patterns (65 to 74 year olds)

|  | **No MM** | **Eye** | **Unspecific (Complex)** | **Cardiovascular** | **Mental health** | **Unspecific (Risk)** | **Multisystem** |
| --- | --- | --- | --- | --- | --- | --- | --- |
|  | **N=16,066** | **N=11,321** | **N=32,698** | **N=8,973** | **N=6,089** | **N=37,297** | **N=8,016** |
| **Age** | 69.1 [±2.8] | 70.5 [±2.8] | 69.6 [±2.8] | 70.3 [±2.9] | 69.4 [±2.9] | 69.6 [±2.8] | 70.2 [±2.9] |
| **Sex (female)** | 48.3 (7,763) | 58.4 (6,609) | 56.9 (18,615) | 37.0 (3,323) | 69.1 (4,207) | 46.2 (17,221) | 75.3 (6,033) |
| **Number of conditions** | 0.7 [±0.4] | 4.9 [±1.9] | 4.1 [±1.7] | 7.7 [±2.7] | 6.4 [±2.3] | 3.7 [±1.6] | 9.3 [±2.4] |
| **eGFR** | 72 [±10] | 70 [±11] | 71 [±11] | 66 [±15] | 72 [±12] | 70 [±12] | 71 [±12] |
| **eGFR <60** | 11.6 (1,859) | 15.6 (1,763) | 14.2 (4,654) | 29.2 (2,621) | 14.4 (875) | 16.3 (6,095) | 16.6 (1,333) |
| **Outcome (Incident A2 or A3)** |  |  |  |  |  |  |  |
| Administrative censoring | 76.9 (12,358) | 80.0 (9,055) | 73.9 (24,172) | 48.9 (4,384) | 74.6 (4,541) | 69.9 (26,089) | 77.4 (6,201) |
| Incident A2 | 6.6 (1,068) | 7.4 (834) | 7.3 (2,375) | 16.5 (1,482) | 6.3 (381) | 11.9 (4,441) | 7.5 (602) |
| Death | 11.5 (1,843) | 9.8 (1,106) | 15.1 (4,943) | 31.3 (2,811) | 15.7 (959) | 13.7 (5,127) | 12.2 (981) |
| RRT | 0.0 (3) | 0.0 (0) | 0.0 (5) | 0.2 (14) | 0.0 (0) | 0.0 (14) | 0.0 (2) |
| Emigration | 4.9 (794) | 2.9 (326) | 3.7 (1,203) | 3.1 (282) | 3.4 (208) | 4.4 (1,626) | 2.9 (230) |
| **Outcome (Incident A3)** |  |  |  |  |  |  |  |
| Administrative censoring | 80.8 (12,982) | 84.0 (9,511) | 77.7 (25,400) | 54.9 (4,928) | 77.8 (4,739) | 76.5 (28,520) | 81.4 (6,523) |
| Incident A2 | 1.5 (246) | 1.7 (189) | 1.8 (590) | 4.2 (380) | 1.4 (83) | 2.5 (924) | 1.9 (150) |
| Death | 12.6 (2,017) | 11.3 (1,276) | 16.7 (5,466) | 37.2 (3,341) | 17.3 (1,051) | 16.4 (6,129) | 13.7 (1,100) |
| RRT | 0.0 (3) | 0.0 (4) | 0.0 (15) | 0.2 (19) | 0.0 (1) | 0.1 (22) | 0.0 (2) |
| Emigration | 5.1 (818) | 3.0 (341) | 3.8 (1,227) | 3.4 (305) | 3.5 (215) | 4.6 (1,702) | 3.0 (241) |
| Data are presented as mean [±SD] for continuous measures, and % (n) for categorical measures. | | | | | | | |

**Table S11.** Conditions characterizing each multimorbidity pattern among 18-64 year olds (shaded meet both overexpression criteria).

| **Multimorbidity**  **Pattern** | **Disease** | **Exclusivity (%)** | **Observed/expected**  **Ratio** | **Prevalence (%)** |
| --- | --- | --- | --- | --- |
| **Unspecific** | Inflammatory bowel diseases | 76 | 2 | 6,5 |
| **(n=117,315)** | Inflammatory arthropathies | 67 | 1,7 | 12,4 |
|  | Osteoporosis | 67 | 1,7 | 4,1 |
|  | Autoimmune diseases | 60 | 1,6 | 10,4 |
|  | Blood and blood forming organ diseases | 61 | 1,6 | 3,5 |
|  | Allergy | 55 | 1,4 | 14,8 |
|  | Anemia | 51 | 1,3 | 16,6 |
|  | Chronic infectious diseases | 49 | 1,3 | 6,2 |
|  | Chronic pancreas, biliary tract and gallbladder diseases | 49 | 1,3 | 4,9 |
|  | Colitis and related diseases | 48 | 1,3 | 17,6 |
|  | Ear, nose, throat diseases | 50 | 1,3 | 12,6 |
|  | Other metabolic diseases | 48 | 1,3 | 5,6 |
|  | Solid neoplasms | 50 | 1,3 | 9,6 |
|  | Asthma | 48 | 1,2 | 13,5 |
|  | Esophagus, stomach and duodenum diseases | 47 | 1,2 | 11,6 |
|  | Migraine and facial pain syndromes | 45 | 1,2 | 14,5 |
|  | Other genitourinary diseases | 47 | 1,2 | 13,7 |
|  | Prostate diseases | 46 | 1,2 | 4,7 |
|  | Venous and lymphatic diseases | 47 | 1,2 | 3,3 |
|  | Dorsopathies | 44 | 1,1 | 10,9 |
|  | Other musculoskeletal and joint diseases | 44 | 1,1 | 24,6 |
|  | Peripheral neuropathy | 43 | 1,1 | 7,7 |
|  | Thyroid diseases | 44 | 1,1 | 13,2 |
|  | Deafness, hearing impairment | 38 | 1 | 4,3 |
|  | Osteoarthritis and other degenerative joint diseases | 39 | 1 | 9,3 |
|  | Other neurological diseases | 40 | 1 | 2,9 |
|  | Other eye diseases | 34 | 0,9 | 6 |
|  | COPD, emphysema, chronic bronchitis | 31 | 0,8 | 2,5 |
|  | Cataract and other lens diseases | 27 | 0,7 | 2,1 |
| **Cardiometabolic** | Dyslipidemia | 85 | 2,9 | 31,3 |
| **(n=90,184)** | Ischemic heart disease | 74 | 2,5 | 13,7 |
|  | Diabetes | 69 | 2,4 | 37,8 |
|  | Hypertension | 70 | 2,4 | 74,6 |
|  | Cerebrovascular disease | 67 | 2,3 | 5,7 |
|  | Cataract and other lens diseases | 48 | 1,6 | 4,9 |
|  | Obesity | 45 | 1,5 | 15,7 |
|  | Other eye diseases | 38 | 1,3 | 8,8 |
|  | Osteoarthritis and other degenerative joint diseases | 34 | 1,2 | 10,7 |
|  | Prostate diseases | 34 | 1,2 | 4,5 |
|  | COPD, emphysema, chronic bronchitis | 33 | 1,1 | 3,5 |
|  | Deafness, hearing impairment | 28 | 1 | 4,1 |
|  | Chronic infectious diseases | 27 | 0,9 | 4,5 |
|  | Solid neoplasms | 26 | 0,9 | 6,5 |
| **Mental health** | Depression and mood diseases | 75 | 2,9 | 64,6 |
| **(n=79,343)** | Neurotic, stress-related and somatoform diseases | 73 | 2,8 | 83,9 |
|  | Other psychiatric and behavioral diseases | 54 | 2,1 | 21,3 |
|  | Sleep disorders | 43 | 1,7 | 15,1 |
|  | Migraine and facial pain syndromes | 33 | 1,3 | 15,9 |
|  | Allergy | 29 | 1,1 | 11,4 |
|  | Anemia | 29 | 1,1 | 14,2 |
|  | Colitis and related diseases | 30 | 1,1 | 16,2 |
|  | Ear, nose, throat diseases | 26 | 1 | 9,7 |
|  | Esophagus, stomach and duodenum diseases | 26 | 1 | 9,5 |
|  | Other metabolic diseases | 25 | 1 | 4,4 |
| **Neuro-Musculoskeletal** | Peripheral neuropathy | 34 | 5,6 | 39 |
| **(n=18,645)** | Dorsopathies | 28 | 4,6 | 44,7 |
|  | COPD, emphysema, chronic bronchitis | 25 | 4,2 | 12,9 |
|  | Osteoarthritis and other degenerative joint diseases | 23 | 3,8 | 34,8 |
|  | Cataract and other lens diseases | 21 | 3,4 | 10,3 |
|  | Other neurological diseases | 19 | 3,1 | 8,6 |
|  | Osteoporosis | 18 | 3 | 6,9 |
|  | Sleep disorders | 18 | 2,9 | 26 |
|  | Other eye diseases | 16 | 2,7 | 18,4 |
|  | Other musculoskeletal and joint diseases | 17 | 2,7 | 59,1 |
|  | Deafness, hearing impairment | 16 | 2,5 | 11 |
|  | Esophagus, stomach and duodenum diseases | 15 | 2,5 | 24,2 |
|  | Cerebrovascular disease | 15 | 2,4 | 6 |
|  | Depression and mood diseases | 15 | 2,4 | 54,5 |
|  | Ischemic heart disease | 15 | 2,4 | 13,3 |
|  | Venous and lymphatic diseases | 15 | 2,4 | 6,5 |
|  | Asthma | 14 | 2,3 | 25,1 |
|  | Obesity | 14 | 2,3 | 24 |
|  | Other genitourinary diseases | 14 | 2,3 | 25,9 |
|  | Chronic pancreas, biliary tract and gallbladder diseases | 13 | 2,2 | 8,5 |
|  | Migraine and facial pain syndromes | 13 | 2,1 | 25,7 |
|  | Neurotic, stress-related and somatoform diseases | 13 | 2,1 | 63,8 |
|  | Colitis and related diseases | 12 | 2 | 28,4 |
|  | Ear, nose, throat diseases | 12 | 2 | 19,7 |

**Table S12.** Description of patterns (18 to 64 year olds)

|  | **No MM** | **Cardiometabolic** | **Mental health** | **Unspecific** | **Neuro-MSK** |
| --- | --- | --- | --- | --- | --- |
|  | **N=164,634** | **N=90,184** | **N=79,343** | **N=117,315** | **N=18,645** |
| **Age** | 41.4 [±12.8] | 54.0 [±9.3] | 40.3 [±12.3] | 45.3 [±12.9] | 52.6 [±9.6] |
| **Sex (female)** | 48.8 (80,390) | 41.4 (37,295) | 69.4 (55,079) | 61.2 (71,829) | 71.5 (13,327) |
| **Number of conditions** | 0.6 [±0.5] | 3.6 [±1.7] | 4.0 [±1.7] | 3.3 [±1.5] | 8.5 [±2.3] |
| **eGFR** | 89 [±14] | 84 [±14] | 91 [±14] | 89 [±15] | 86 [±14] |
| **eGFR <60** | 1.1 (1,848) | 3.5 (3,178) | 1.1 (881) | 2.0 (2,359) | 3.0 (567) |
| **Outcome (Incident A2 or A3)** |  |  |  |  |  |
| Administrative censoring | 84.9 (139,835) | 78.4 (70,692) | 85.4 (67,749) | 83.8 (98,282) | 82.8 (15,429) |
| Incident A2 | 2.5 (4,055) | 8.7 (7,853) | 3.0 (2,345) | 4.5 (5,265) | 6.0 (1,122) |
| Death | 1.3 (2,219) | 5.5 (4,946) | 2.2 (1,725) | 3.5 (4,062) | 5.6 (1,050) |
| RRT | 0.0 (11) | 0.0 (17) | 0.0 (2) | 0.0 (21) | 0.0 (2) |
| Emigration | 11.2 (18,514) | 7.4 (6,676) | 9.5 (7,522) | 8.3 (9,685) | 5.6 (1,042) |
| **Outcome (Incident A3)** |  |  |  |  |  |
| Administrative censoring | 86.7 (142,676) | 83.9 (75,699) | 87.5 (69,407) | 86.6 (101,620) | 86.6 (16,146) |
| Incident A2 | 0.5 (854) | 1.8 (1,644) | 0.6 (469) | 1.1 (1,242) | 1.3 (235) |
| Death | 1.4 (2,368) | 6.4 (5,797) | 2.3 (1,808) | 3.8 (4,478) | 6.4 (1,188) |
| RRT | 0.0 (21) | 0.1 (60) | 0.0 (11) | 0.0 (39) | 0.0 (5) |
| Emigration | 11.4 (18,715) | 7.7 (6,984) | 9.6 (7,648) | 8.5 (9,936) | 5.7 (1,071) |
| Data are presented as mean [±SD] for continuous measures, and % (n) for categorical measures. | | | | | |

**Table S13**. Numbers remaining at risk over a 10-year period

| **75+ year olds, outcome A2+** | | | | | | | | | |
| --- | --- | --- | --- | --- | --- | --- | --- | --- | --- |
|  |  | **Vascular** | **Unspecific**  **RF** | **Cardiovascular** | **Multisystem** | **Eye** | **Unspecific**  **Complex** | **Dementia** | **No MM** |
| **Years** | **0** | 2000 | 18626 | 10559 | 8647 | 16047 | 18785 | 4823 | 5502 |
|  | **2** | 1418 | 15217 | 7041 | 6245 | 12252 | 15100 | 3378 | 4771 |
|  | **4** | 936 | 11837 | 4383 | 4039 | 8458 | 11770 | 2123 | 4024 |
|  | **6** | 588 | 8688 | 2569 | 2406 | 5331 | 8813 | 1204 | 3279 |
|  | **8** | 327 | 5786 | 1339 | 1204 | 3010 | 6188 | 630 | 2530 |
|  | **10** | 175 | 3728 | 676 | 611 | 1691 | 4137 | 301 | 1863 |

| **75+ year olds, outcome A3** | | | | | | | | | |
| --- | --- | --- | --- | --- | --- | --- | --- | --- | --- |
|  |  | **Vascular** | **Unspecific**  **RF** | **Cardiovascular** | **Multisystem** | **Eye** | **Unspecific**  **Complex** | **Dementia** | **No MM** |
| **Years** | **0** | 2000 | 18626 | 10559 | 8647 | 16047 | 18785 | 4823 | 5502 |
|  | **2** | 1501 | 15833 | 7412 | 6424 | 12609 | 15569 | 3468 | 4861 |
|  | **4** | 1049 | 12785 | 4819 | 4288 | 8977 | 12451 | 2213 | 4177 |
|  | **6** | 674 | 9678 | 2920 | 2614 | 5802 | 9514 | 1271 | 3461 |
|  | **8** | 396 | 6701 | 1577 | 1338 | 3356 | 6806 | 672 | 2718 |
|  | **10** | 225 | 4449 | 826 | 700 | 1928 | 4599 | 324 | 2036 |

| **65-74 year olds, outcome A2+** | | | | | | | | |
| --- | --- | --- | --- | --- | --- | --- | --- | --- |
|  |  | **Cardiovascular** | **Unspecific**  **RF** | **Multisystem** | **Eye** | **Mental health** | **Unspecific**  **Complex** | **No MM** |
| **Years** | **0** | 8973 | 37297 | 8016 | 11321 | 6089 | 32698 | 16066 |
|  | **2** | 6907 | 31558 | 6308 | 9073 | 4747 | 27020 | 14010 |
|  | **4** | 5220 | 25698 | 4571 | 6548 | 3523 | 21983 | 12043 |
|  | **6** | 3745 | 20109 | 3071 | 4356 | 2464 | 17210 | 10067 |
|  | **8** | 2488 | 14428 | 1829 | 2460 | 1547 | 12505 | 8027 |
|  | **10** | 1588 | 10353 | 1140 | 1565 | 1027 | 9133 | 6424 |

| **65-74 year olds, outcome A3** | | | | | | | | |
| --- | --- | --- | --- | --- | --- | --- | --- | --- |
|  |  | **Cardiovascular** | **Unspecific**  **RF** | **Multisystem** | **Eye** | **Mental health** | **Unspecific**  **Complex** | **No MM** |
| **Years** | **0** | 8973 | 37297 | 8016 | 11321 | 6089 | 32698 | 16066 |
|  | **2** | 7295 | 32352 | 6441 | 9255 | 4836 | 27549 | 14163 |
|  | **4** | 5778 | 27122 | 4781 | 6855 | 3659 | 22763 | 12312 |
|  | **6** | 4339 | 21817 | 3311 | 4680 | 2594 | 18060 | 10375 |
|  | **8** | 3001 | 16216 | 2033 | 2757 | 1658 | 13342 | 8418 |
|  | **10** | 2004 | 12106 | 1314 | 1802 | 1131 | 9944 | 6857 |

| **18-64 year olds, outcome A2+** | | | | | | |
| --- | --- | --- | --- | --- | --- | --- |
|  |  | **Cardiometabolic** | **Neuro-musculoskeletal** | **Unspecific** | **Mental health** | **No MM** |
| **Years** | **0** | 90184 | 18645 | 117315 | 79343 | 164634 |
|  | **2** | 74180 | 14752 | 95289 | 60639 | 135308 |
|  | **4** | 59723 | 11231 | 76482 | 45570 | 110965 |
|  | **6** | 47038 | 7932 | 57942 | 31566 | 86368 |
|  | **8** | 35375 | 5008 | 41116 | 19874 | 64146 |
|  | **10** | 26982 | 3496 | 31052 | 13966 | 50486 |
| **18-64 year olds, outcome A3** | | | | | | |
|  |  | **Cardiometabolic** | **Neuro-musculoskeletal** | **Unspecific** | **Mental health** | **No MM** |
| **Years** | **0** | 90184 | 18645 | 117315 | 79343 | 164634 |
|  | **2** | 75735 | 15045 | 96814 | 61380 | 136314 |
|  | **4** | 62250 | 11653 | 78589 | 46541 | 112424 |
|  | **6** | 50075 | 8385 | 60117 | 32494 | 87956 |
|  | **8** | 38650 | 5423 | 43117 | 20620 | 65698 |
|  | **10** | 30319 | 3867 | 32922 | 14593 | 52081 |

**Figure S1**. Study flowchart


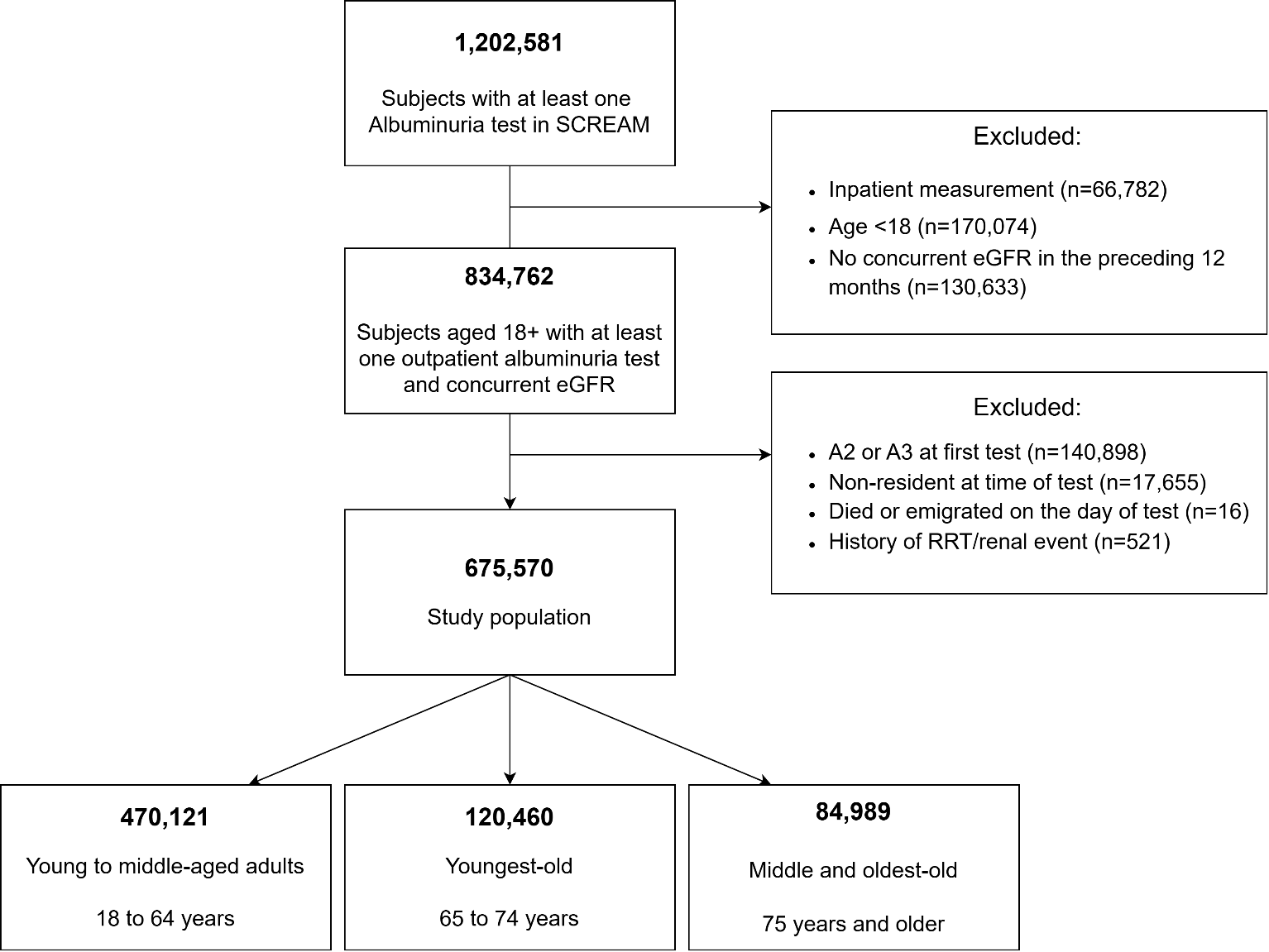


**Figure S2.** Interactions between multimorbidity patterns and sex (panels A,C,E) and eGFR (Panels B,D,F) with the outcome of albuminuria


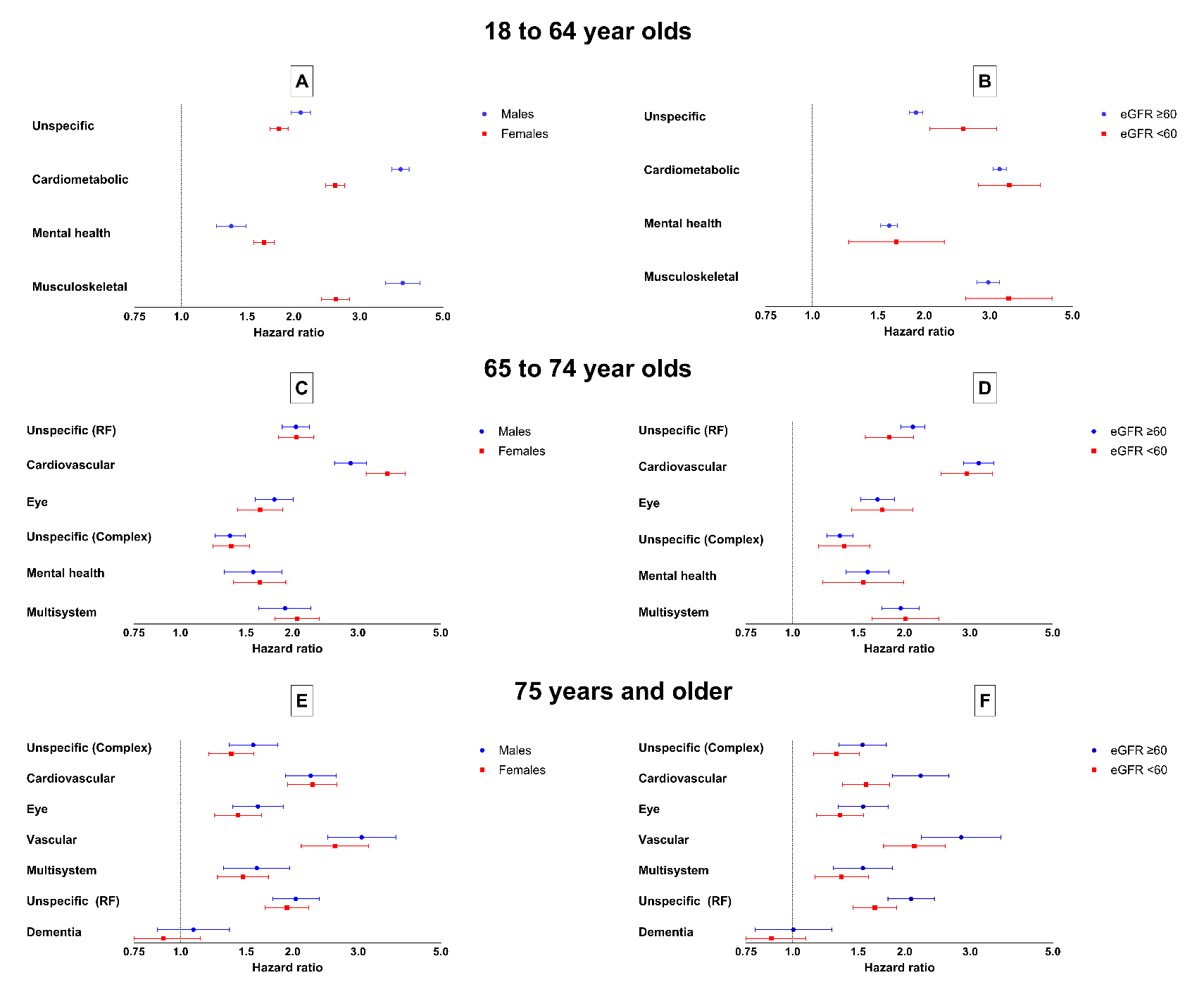
 Note: reference group for all reported estimates is the group without multimorbidity

Abbreviations: eGFR, estimated glomerular filtration rate; RF, risk factors.
